# Gastrointestinal axis in post-traumatic sepsis: from molecular mechanisms to translational perspectives

**DOI:** 10.3389/fimmu.2026.1855955

**Published:** 2026-07-17

**Authors:** Xiang-yu Liu, Tian Liu, Jia-ke Chai, Yu-shou Wu, Hong-sheng Liu, Yi-rui Qu, Hui Zhou, Cheng-feng Xu, Yun-fei Chi

**Affiliations:** Fourth Medical Centre, Chinese PLA General Hospital, Beijing, China

**Keywords:** autophagy, biomarkers, gastrointestinal axis, organ crosstalk, post-traumatic sepsis

## Abstract

Post-traumatic sepsis is the leading cause of late mortality in intensive care units. While the concept of “gut-origin sepsis” has evolved over the past three decades, the stomach has largely been overlooked as an active contributor. This review synthesizes current evidence and proposes a novel “Gastrointestinal Axis” (GIA) integrating gastric and intestinal dysfunction as interconnected drivers of post-traumatic sepsis, with gastric autophagy as a proposed regulatory node. The mechanistic data supporting this framework derive primarily from intestinal epithelial cells and general sepsis models; direct evidence for gastric mucosal autophagy in human post-traumatic sepsis remains limited, and the causal inference that gastric autophagy failure initiates downstream intestinal injury remains a hypothesis requiring further validation. A comprehensive literature search of PubMed, Web of Science, and Scopus was conducted to identify relevant peer-reviewed studies on gut barrier dysfunction, dysbiosis, autophagy, and immune responses in post-traumatic sepsis. The central mechanistic framework involves histone deacetylase 5 (HDAC5) upregulation silencing ghrelin, thereby impairing gastric autophagy—a proposed regulatory node in the GIA—which reduces E2F1-mediated NF-κB suppression and impairs intestinal barrier integrity. Parallel protective pathways include PLK1-mTOR-regulated autophagy and SIRT3-mediated mitochondrial protection. This gastric dysfunction may propagate to the intestine, where dysbiosis with loss of obligate anaerobes and overgrowth of Enterobacteriaceae creates a “pathobiome” that potentially amplifies systemic inflammation. Bidirectional communication occurs via lymphatic, humoral, cellular, and neural routes. Emerging biomarkers such as intestinal fatty acid-binding protein, D-lactate, citrulline, and the Acute Gastrointestinal Injury grading system enable multimodal risk stratification. Early enteral nutrition (OR 0.36) and synbiotics (RR 0.61) show promise; preclinical data support HDAC5 inhibitors, ghrelin restoration, and teprenone as promising adjuncts for preserving GIA integrity. The GIA concept reframes gastric and intestinal protection as an integrated therapeutic strategy and provides a new conceptual foundation for preventing post-traumatic sepsis and guiding biomarker-driven mechanism-based interventions.

## 1 Introduction

Post-traumatic sepsis remains a formidable challenge in modern intensive care units (ICUs), contributing substantially to morbidity, mortality, and healthcare costs (1, 2). Despite advances in resuscitation, antibiotic therapy, and organ support, the incidence of sepsis following major trauma, burns, and surgery has increased in recent decades, with mortality rates of 30%–40% (3, 4). A striking epidemiological shift has occurred: while early deaths from refractory shock have declined, late-onset sepsis, occurring days to weeks after the initial injury, now predominates and is associated with immunosuppression, multidrug-resistant pathogens, and progressive organ failure (5).

The gastrointestinal tract has long been implicated in this process; over the last five decades, conceptual frameworks have evolved from the “gut hypothesis of sepsis,” emphasizing bacterial translocation (6–8), to the gut lymph hypothesis, which highlights the role of mesenteric lymphatics in transporting gut-derived factors into systemic circulation (9–11). These advances have established the intestine as a cytokine-generating organ driving systemic inflammation (12, 13). However, these models have largely overlooked the contribution of the proximal gastrointestinal tract, particularly the stomach.

The proximal gastrointestinal tract has largely been viewed as a passive conduit for enteral nutrition or as a source of stress-related bleeding. However, emerging evidence has shown that gastric injury and dysfunction play an active role in the pathogenesis of post-traumatic sepsis. Gastric ischemia, impaired mucosal defense, and dysregulated autophagy, a critical cellular mechanism for maintaining homeostasis under stress, may initiate a cascade that extends to the distal intestine and beyond (14–16). The gastric hormone ghrelin has emerged as a key regulator of autophagy via the microRNA 143 (miR-143)/autophagy-related gene 2B (ATG2B) pathway to protect the intestinal barrier (17, 18). This protective axis is counter-regulated by histone deacetylase 5 (HDAC5), which transcriptionally inhibits ghrelin, leading to nuclear factor kappa-B (NF-κB) activation and intestinal inflammation (19).

Of all traumatic injuries, severe burns represent an extreme form of trauma that uniquely illustrates the progression from initial shock to late-onset sepsis. In the early phase, immediately post-burn, patients develop burn shock characterized by profound hypovolemia, gastrointestinal hypoperfusion, and mucosal ischemia (20). Preclinical studies have shown that gastroprotective interventions initiated during this acute phase, such as teprenone-supplemented oral rehydration, can preserve gastric integrity and improve survival (21, 22), raising the possibility that early modulation of gastric function may have enduring effects that extend beyond shock reversal to mitigate subsequent septic complications.

These observations highlight the need for an integrated framework that links gastric and intestinal function. We propose the “Gastrointestinal Axis” (GIA), a conceptual model that positions the stomach as an active regulator of distal intestinal homeostasis, with gastric autophagy proposed as a key mechanistic node. In this model, gastric injury and autophagic dysfunction may initiate a cascade that can propagate to the intestine, where barrier disruption, dysbiosis, and immune activation amplify systemic inflammation, ultimately contributing to the progression to sepsis. This framework builds upon established organ-axis paradigms, including the gut–liver, gut–lung, and gut–brain axes, as well as the emerging gut–muscle and gut–temperature axes (23–29). It should be noted that direct evidence for autophagy dysregulation within the gastric mucosa in human post-traumatic sepsis remains limited; the mechanistic data supporting this framework derive primarily from intestinal epithelial cells and general sepsis models. The causal inference that gastric autophagy failure initiates downstream intestinal injury therefore remains a plausible hypothesis requiring further validation.

The GIA framework differs from existing paradigms in three key aspects. First, whereas the gut-origin sepsis and gut–lymph hypotheses position the intestine as the primary source of inflammatory mediators, the GIA explicitly incorporates the stomach as an active upstream regulator of distal intestinal homeostasis. Second, while the gut–liver, gut–lung, and gut–brain axes describe parallel organ-to-organ communication pathways involving the intestine, the GIA posits a sequential, feed-forward cascade in which gastric dysfunction precedes and amplifies intestinal injury, rather than treating gastric and intestinal dysfunction as separate events. Third, the GIA identifies gastric autophagy—under the hierarchical control of the HDAC5–ghrelin–miR-143–ATG2B axis—as the specific molecular node linking the stomach and intestine, a mechanistic link not captured by prior conceptual models. It is important to acknowledge that individual components of the GIA—including ghrelin-mediated autophagy, the HDAC5–ghrelin signaling axis, and intestinal barrier dysfunction in sepsis—have been described in prior publications. The novelty of the GIA therefore lies not in the discovery of any single pathway, but in the conceptual synthesis that positions the stomach as the upstream initiator of a sequential cascade, with gastric autophagy as the proposed mechanistic bridge linking proximal gastric injury to distal intestinal failure. This integrated, stomach-centric perspective represents a departure from existing frameworks, which have largely treated gastric and intestinal dysfunction as parallel or independent phenomena rather than as causally connected components of a single pathologic axis.

Post-traumatic sepsis differs from spontaneous sepsis in several clinically important respects. Unlike community-acquired sepsis, which typically arises from a single infectious focus, post-traumatic sepsis develops against a background of profound tissue injury, ischemia–reperfusion damage, and a dysregulated immune response. The early post-traumatic period is characterized by severe splanchnic hypoperfusion, which directly compromises gastric mucosal integrity and ghrelin secretion (15, 16, 20). This initial insult is followed by a prolonged phase of immunosuppression, during which patients are highly vulnerable to nosocomial infections and the emergence of a pathogenic gut microbiome—a transition that underlies the high incidence of late-onset sepsis (5). Furthermore, the predictable temporal progression from injury through shock to resuscitation offers a unique window for early gastroprotective intervention, as demonstrated by preclinical studies showing that teprenone-supplemented oral rehydration during burn shock preserves gastric integrity and improves survival (21, 22). Importantly, patients with trauma and burns are often previously healthy young adults without significant comorbidities (1–3), and the defined time-zero of injury enables controlled study of sequential pathophysiological events that is not feasible in spontaneous sepsis. These trauma-specific features position post-traumatic sepsis as a distinct clinical entity in which the GIA may play a particularly prominent role. Nevertheless, the core mechanisms of the GIA—ghrelin-mediated autophagy, intestinal barrier disruption, and dysbiosis-driven systemic inflammation—are likely relevant to sepsis of all etiologies, though the severity and timing of gastric involvement may differ.

**Figure 1:**
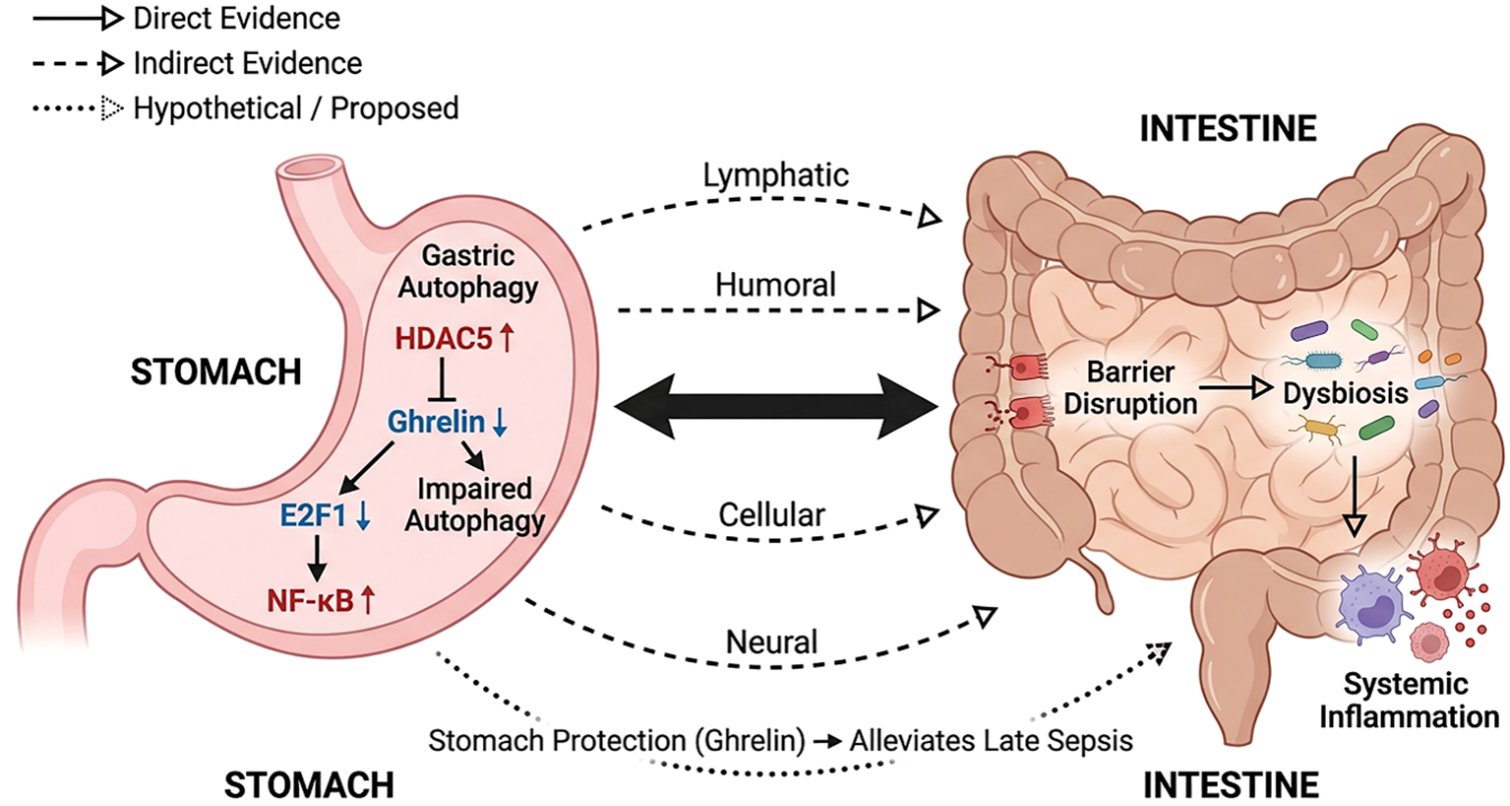
GIA in post-traumatic sepsis. Traumatic injury induces gastric and intestinal hypoperfusion, leading to HDAC5 upregulation in the gastric mucosa. HDAC5 transcriptionally suppresses ghrelin, which impairs autophagy via the miR-143–ATG2B pathway and removes E2F1-mediated inhibition of NF-κB, unleashing pro-inflammatory cytokine production. Gastric-derived signals may propagate to the intestine via lymphatic, humoral, cellular, and neural routes, where barrier disruption, dysbiosis (loss of obligate anaerobes, Enterobacteriaceae overgrowth), and immune dysregulation amplify systemic inflammation. Solid lines indicate experimentally supported pathways; dashed lines indicate indirectly supported links; dotted lines indicate hypothetical or proposed links. ATG2B, autophagy-related gene 2B; E2F1, E2F transcription factor 1; GIA, Gastrointestinal Axis; HDAC5, histone deacetylase 5; NF-κB, nuclear factor kappa-B.

In this review, we synthesize current evidence supporting the GIA as a conceptual foundation for understanding post-traumatic sepsis (**Figure 1**). We focus on recently uncovered converging molecular pathways in gastric autophagy, including the endogenous HDAC5–ghrelin–miR-143–ATG2B axis, and its downstream effects on intestinal barrier integrity. We also examine pharmacologically inducible mechanisms, such as those mediated by teprenone, which may use parallel autophagic mechanisms to preserve GIA integrity. By framing gastric and intestinal protection as an integrated therapeutic strategy, the GIA framework provides a new paradigm for identifying novel biomarkers and developing mechanism-based interventions.

## 2 Methods

### 2.1 Search strategy

A comprehensive literature search was conducted in PubMed, Web of Science, and Scopus from database inception to December 31, 2025. The search combined terms related to three domains: (1) trauma, burns, shock, and sepsis; (2) gastrointestinal tract, stomach, intestine, gut barrier, and autophagy; and (3) microbiome, microbiota, biomarkers, and therapy. The initial search yielded 9,210 records from PubMed, 3,980 from Web of Science, and 27,445 from Scopus. The full search strategy and yield for each database are provided in **Supplementary Table 1**. Reference lists of included studies and relevant reviews were hand-searched for additional eligible publications.

### 2.2 Study selection

Two reviewers (X.-Y.L. and T.L.) independently screened titles and abstracts, followed by full-text review of potentially eligible studies. Any disagreements were resolved through discussion with a third reviewer (Y.-F.C.). Studies were selected based on their relevance to gastrointestinal dysfunction in the context of trauma, burns, shock, or sepsis, with priority given to peer-reviewed original research, clinical trials, meta-analyses, and systematic reviews. Studies focusing exclusively on non-sepsis gastrointestinal diseases without relevance to post-traumatic sepsis were excluded. Given the narrative nature of this review, formal quality assessment and quantitative synthesis were not performed.

## 3 The intestinal microenvironment in sepsis: barrier, microbiome, and immunity

The intestine is not merely a passive organ for nutrient absorption but instead a complex ecosystem essential for host survival. Its integrity depends on three interdependent components—the epithelial barrier, gut microbiome, and mucosal immune system—which maintain homeostasis under physiological conditions and are profoundly disrupted in sepsis (30). This section provides essential background on the intestinal microenvironment in sepsis.

### 3.1 The intestinal epithelial barrier: structure and dysfunction

The intestinal epithelial barrier comprises a single layer of cells connected by tight junctions (TJs) that regulate paracellular permeability (31, 32). During sepsis, this barrier is rapidly disrupted through multiple mechanisms (33). Within hours of sepsis onset, TJ proteins are redistributed: claudin-2 and junctional adhesion molecule are upregulated, whereas claudin-5 and occludin are reduced, as observed in both cecal ligation and puncture (CLP) and *Pseudomonas aeruginosa* pneumonia models (34, 35). These changes are mediated by myosin light chain kinase, which phosphorylates the regulatory light chain and induces contraction of the perijunctional actin-myosin ring (36, 37).

Intestinal epithelial cell (IEC) apoptosis is also markedly upregulated in sepsis and is driven by both death receptor and mitochondrial pathways (38, 39). Hotchkiss et al. (40) documented increased IEC apoptosis in patients with sepsis at autopsy, which was correlated with disease severity. In experimental models, transgenic overexpression of the anti-apoptotic protein Bcl-2 in the intestinal epithelium improves survival in murine sepsis models, establishing a causal link between IEC apoptosis and mortality (41, 42).

The mucus layer, composed primarily of mucin 2, forms a physical barrier that separates luminal microbes from the epithelium (43). In critical illness, mucus thickness diminishes, and luminal coverage becomes patchy, facilitating bacterial–epithelial contact (44–46). In addition to these structural changes, oxidative stress plays a critical role in tight junction disruption during sepsis. Sepsis-induced generation of reactive oxygen species (ROS) directly oxidizes tight junction proteins such as occludin and claudins, leading to their conformational change, redistribution, and proteasomal degradation. ROS also activate redox-sensitive signaling pathways, including mitogen-activated protein kinase (MAPK) and NF-κB, which transcriptionally downregulate tight junction protein expression and promote epithelial apoptosis. Furthermore, lipid peroxidation products such as 4-hydroxynonenal form covalent adducts with tight junction proteins, impairing their assembly and functional integrity. Collectively, these structural changes comprise the first line of defense in barrier integrity and set the stage for systemic inflammatory propagation.

### 3.2 The gut microbiome: from homeostasis to pathobiome

The human gut harbors trillions of bacteria that support host health through nutrient metabolism, colonization resistance, and immune education (47–49). In sepsis, this balanced ecosystem is rapidly disrupted. Within hours of insult, protective anaerobes decline, and pathogenic Proteobacteria such as *Escherichia*, *Klebsiella*, and *Pseudomonas* expand. This shift results in a low-diversity, multidrug-resistant-dominated “pathobiome” strongly linked to late-onset sepsis and mortality (5, 50–55).

The clinical relevance of dysbiosis in critically ill patients has been shown in multiple large cohorts. Schlechte et al. (56) reported that progressive *Enterobacteriaceae* enrichment in critically ill patients was associated with a 6.8-fold increased odds of nosocomial infection. Chanderraj et al. (57) further provided causal evidence from a large cohort of 3032 mechanically ventilated patients, showing that anti-anaerobic antibiotic exposure increased the risk of ventilator-associated pneumonia (VAP) by 24%. This finding was supported by experimental murine models in which depletion of anaerobic bacteria impaired pathogen clearance. Host factors also influence microbiome resilience; for instance, women exhibit greater microbiome resilience (58, 59).

In addition to bacterial communities, the gut mycobiome is also profoundly altered in sepsis. Park et al. (60) reported that patients with sepsis and trauma exhibit persistent fungal dysbiosis, with *Candida* species comprising > 95% of the fungal community, creating an ecological niche that favors invasive candidiasis.

The gut microbiota produce metabolites that influence host physiology, both locally and systemically. Short-chain fatty acids (SCFAs), including acetate, propionate, and butyrate, are key regulators of local and systemic immunity. They serve as energy substrates for colonocytes, enhance TJ expression, and attenuate inflammation via G-protein coupled receptor 43/109A and HDAC inhibition (61–65). In sepsis, fecal SCFA levels decline and are correlated with disease severity and mortality (66, 67), whereas supplementation with sodium butyrate improves survival in animal models (68, 69). Other metabolites, such as L-valine and indole-3-acetic acid (IAA), also contribute to barrier protection. L-valine levels are inversely correlated with severity and, when supplemented, restore TJ integrity (70). IAA activates the aryl hydrocarbon receptor (AHR) to support epithelial homeostasis and limit neuroinflammation (71). SCFAs also serve as a critical link between the gut microbiome and the gastric endocrine system, as butyrate has been shown to stimulate ghrelin secretion from gastric X/A-like cells, thereby engaging the ghrelin–autophagy axis that protects the intestinal barrier. The consequences of this dysbiosis, including the amplification of systemic inflammation and its role in driving distant organ injury, are central to the pathogenesis of post-traumatic sepsis.

### 3.3 Mucosal immunity: cellular and humoral defenses

Gut-associated lymphoid tissue (GALT) constitutes the largest immune compartment in the body, containing > 80% of all lymphocytes (72). In sepsis, this system is profoundly disrupted. Intraepithelial and lamina propria lymphocytes are depleted (73), and γδ T cells decrease, correlating with increased mortality (74, 75). Paneth cell function is also impaired, leading to decreased secretion of antimicrobial peptides such as α-defensins and facilitating bacterial translocation (76–78). In contrast, antimicrobial peptides such as cathelicidin help preserve barrier integrity (79). Secretory IgA (sIgA), produced by lamina propria plasma cells, normally coats commensal bacteria and prevents epithelial adhesion; however, its production is impaired in critical illness (80–82).

Intestinal alkaline phosphatase (IAP) is a critical innate immune protein present at the mucosal interface (83). Produced by enterocytes, IAP detoxifies lipopolysaccharide (LPS), maintains TJ integrity, and induces autophagy in epithelial cells and macrophages through Toll-like receptor 4 (TLR4)-dependent signaling, thereby directly engaging the autophagic machinery that preserves intestinal homeostasis (84). Notably, IAP activity is modulated by luminal nutrients and microbial metabolites that are, in turn, influenced by gastric hormone signaling, positioning IAP as a potential integrator of proximal and distal gastrointestinal homeostasis.

Lipocalin-2 (Lcn2), also known as neutrophil gelatinase-associated lipocalin, is an innate immune protein produced by the intestinal epithelium and immune cells and exemplifies gut–systemic crosstalk. It limits bacterial growth by sequestering bacterial iron, modulates inflammation, and serves as a clinically established biomarker for sepsis-associated acute kidney injury (AKI) (85). Disruption of these mucosal immune mechanisms compromises intestinal homeostasis and contributes to distal organ dysfunction, highlighting its inextricable link to the functional integrity of the proximal gastrointestinal tract.

## 4 Gastric injury and autophagy: the overlooked proximal gatekeeper

While the intestines bear the brunt of microbial and inflammatory insults during sepsis, the stomach functions as a proximal gatekeeper that regulates the integrity of the entire gastrointestinal tract. Traditionally considered a passive conduit for enteral nutrition, the stomach is an active regulator of distal intestinal homeostasis. Central to this regulatory function is gastric autophagy, a cytoprotective process that preserves cellular integrity under stress conditions (86, 87).

Similar to the intestine, the stomach experiences profound hypoperfusion during shock. Gastric tonometry studies have revealed that persistent intramucosal acidosis is associated with multiple organ dysfunction and increased mortality (88, 89). Gastric barrier dysfunction also shares mechanistic features with intestinal injury, including disruption of tight junction proteins, decreased mucus production, and increased epithelial apoptosis (90).

Collectively, these observations identify the stomach as a vulnerable, yet critical, contributor to the pathogenesis of post-traumatic sepsis. Before discussing the specific molecular pathways, it is important to distinguish three interrelated but distinct processes that are integrated within the GIA framework. Gastric mucosal autophagy refers to autophagic activity within the gastric epithelium, which maintains gastric barrier integrity and supports ghrelin production under stress conditions. Intestinal epithelial autophagy denotes autophagic flux within enterocytes, which preserves tight junction integrity and limits apoptosis during sepsis. Ghrelin-mediated endocrine regulation represents the hormonal link between these two processes: gastric-derived ghrelin acts on intestinal epithelial cells to promote autophagy via the miR-143–ATG2B pathway. Critically, direct evidence for autophagy within the gastric mucosa in post-traumatic sepsis remains limited; most mechanistic data discussed below are derived from intestinal epithelial cells and/or general sepsis models. We present the current state of knowledge transparently, distinguishing direct evidence from inference throughout.

### 4.1 The endogenous signaling axis: HDAC5–ghrelin–miR-143–ATG2B

Autophagy plays a critical role in maintaining cellular homeostasis during sepsis by preserving mitochondrial function, limiting inflammation, and preventing apoptosis (91–93). The gastric hormone ghrelin has emerged as a master regulator of the GIA (94), exerting pleiotropic protective effects. These include the inhibition of pro-inflammatory cytokine release via NF-κB, enhancement of gastrointestinal blood flow by downregulating endothelin-1, and promotion of autophagy in intestinal epithelial cells (95, 96).

Mechanistic studies have begun to define the molecular basis of these effects. In a rat CLP model, Wan et al. (17) provided foundational evidence that ghrelin protects the small intestinal epithelium by enhancing autophagy using autophagy-related proteins, including microtubule-associated protein 1A/1B-light chain 3 (LC3), ATG7, and Beclin-1, with peak activity observed at 8 h post-injury. Further work by Liu et al. (18) showed that ghrelin improves intestinal barrier function by promoting miR-143–ATG2B-mediated autophagy. In both CLP models and LPS-treated IEC-6 cells, ghrelin treatment increased cell viability, upregulated ATG2B and autophagy-related proteins, enhanced autophagosome formation, and reduced miR-143 expression. Dual-luciferase reporter assays confirmed ATG2B as a direct target of miR-143, and overexpression of miR-143 reversed the protective effects of ghrelin, establishing that the ghrelin–miR-143–ATG2B axis is essential for maintaining intestinal barrier integrity. Functionally, this pathway preserves TJ proteins, including claudin-1, occludin, and zonula occludens-1, and reduces systemic markers of intestinal permeability (18). These mechanistic insights into the HDAC5–ghrelin–E2F1–NF-κB signaling cascade have been delineated primarily in rodent CLP models and LPS-treated IEC-6 cells; their operation in human post-traumatic sepsis remains to be validated in prospective clinical studies.

This protective axis is counterregulated at the transcriptional level by HDAC5. Li et al. (19) identified HDAC5 as a key epigenetic regulator that is significantly upregulated in the intestines of patients with sepsis, and is positively correlated with inflammatory cytokines and intestinal fatty acid binding protein (I-FABP). Mechanistically, HDAC5 binds to the ghrelin promoter and suppresses its transcription. Downstream, ghrelin upregulates E2F transcription factor 1 (E2F1) activity, which inhibits NF-κB signaling. HDAC5 knockdown in mice with sepsis improves survival, reduces bacterial burden, attenuates intestinal histopathological damage, and decreases permeability effects recapitulated by the HDAC5 inhibitor LMK-235 (19).

**Figure 2:**
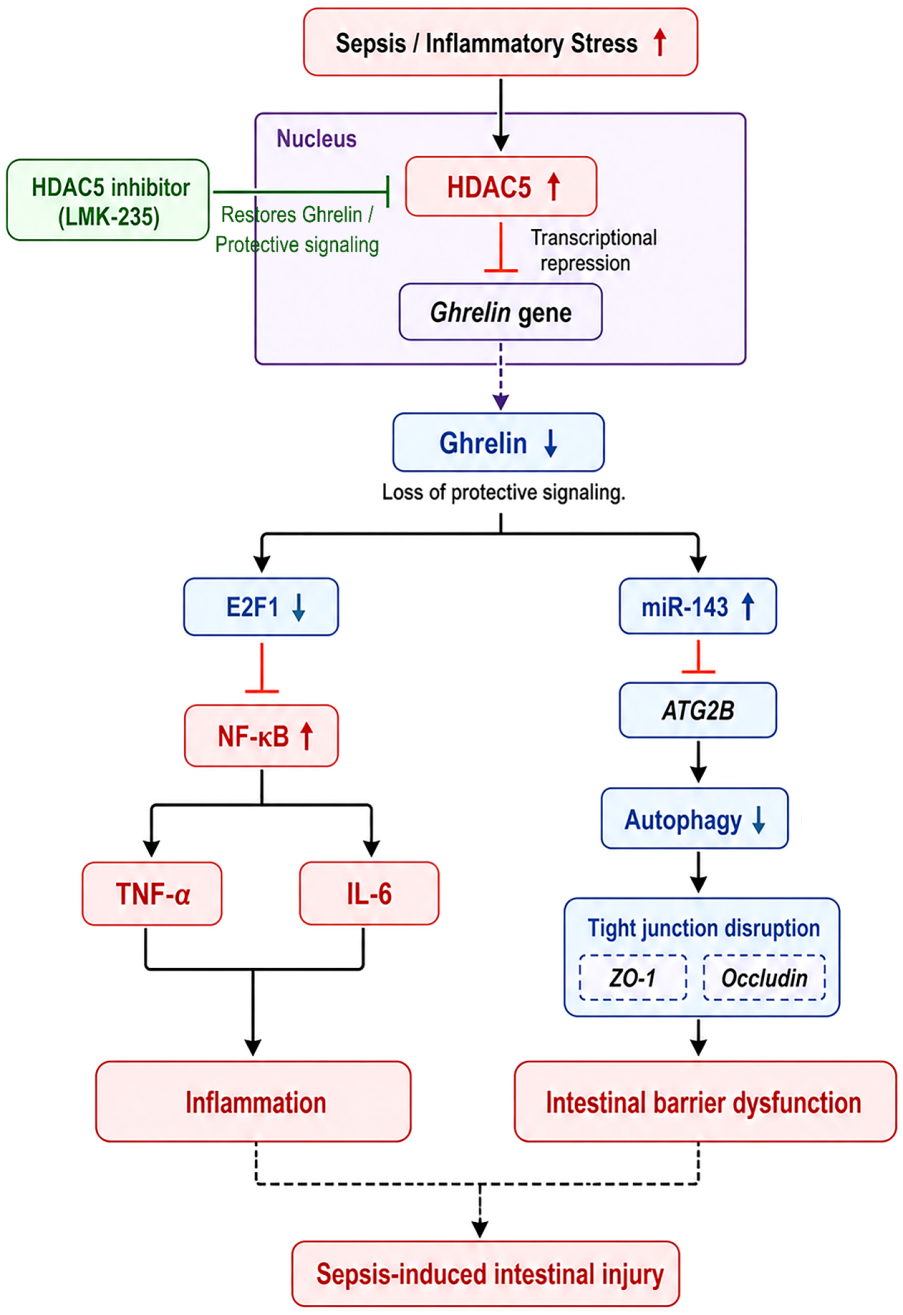
HDAC5–ghrelin–miR-143–ATG2B signaling axis. Sepsis-induced HDAC5 upregulation transcriptionally represses ghrelin expression. Loss of ghrelin signaling reduces miR-143-mediated suppression of ATG2B, impairing autophagic flux and compromising intestinal epithelial barrier integrity as evidenced by decreased zonula occludens-1 (ZO-1) expression. Concurrently, reduced ghrelin diminishes E2F1 activity, removing a critical brake on NF-κB signaling and allowing unchecked production of pro-inflammatory cytokines including tumor necrosis factor-alpha (TNF-α) and interleukin-6 (IL-6). The selective HDAC4/5 inhibitor LMK-235 can restore ghrelin expression and downstream protective effects. ATG2B, autophagy-related gene 2B; E2F1, E2F transcription factor 1; HDAC5, histone deacetylase 5; IL-6, interleukin-6; LMK-235, selective HDAC4/5 inhibitor; miR-143, microRNA-143; NF-κB, nuclear factor kappa-B; TNF-α, tumor necrosis factor-alpha; ZO-1, zonula occludens-1.

These findings define a hierarchical signaling cascade in which sepsis-induced HDAC5 upregulation transcriptionally silences ghrelin, which diminishes E2F1-mediated NF-κB suppression, unleashing pro-inflammatory cytokine production and compromising intestinal barrier integrity. The therapeutic relevance of this axis is further supported by evidence from Zhu et al. (95), revealing that ghrelin–growth hormone secretagogue receptor signaling induces M2 macrophage polarization and alleviates intestinal barrier dysfunction by inactivating E2F1/NF-κB signaling (**Figure 2**).

### 4.2 Parallel protective mechanisms: polo-like kinase 1–mechanistic target of rapamycin and sirtuin 3

In addition to the ghrelin-centered pathway, multiple regulatory nodes contribute to gastric and intestinal protection. Cao et al. (97) identified PLK1 as a critical regulator of intestinal epithelial autophagy through the mTOR pathway. Using conditional PLK1 knock-in mice and LPS-treated intestinal epithelial cells, they revealed that PLK1 overexpression promotes autophagy, reduces apoptosis, and preserves TJ integrity during sepsis. Mechanistically, PLK1 physically interacts with mTOR and negatively regulates its activity. PLK1 knockdown increases mTOR phosphorylation and inhibits autophagy, whereas PLK1 overexpression suppresses mTOR signaling and enhances autophagic flux. The protective effects of PLK1 are autophagy-dependent, as the autophagy inhibitor chloroquine abolishes the beneficial effects on intestinal barrier function, while the autophagy inducer rapamycin enhances them (97). The protective role of PLK1 in intestinal epithelial autophagy has been demonstrated using conditional PLK1 knock-in mice and LPS-treated intestinal epithelial cells; corresponding human data are not yet available.

Xu et al. (98) identified a parallel protective mechanism involving SIRT, a mitochondrial deacetylase that attenuates small intestinal injury by reducing oxidative stress and preserving mitochondrial function. In CLP and endotoxemia models, melatonin treatment upregulates SIRT3, leading to superoxide dismutase 2 deacetylation and activation. SIRT3-mediated mitochondrial protection is associated with enhanced autophagy and reduced apoptosis in intestinal epithelial cells. The specificity of this pathway has been confirmed using selective inhibitors and conditional knockout mice: the SIRT3 inhibitor 3-TYP blocked the protective effects of melatonin, whereas intestinal-specific SIRT3 knockout mice exhibited increased susceptibility to sepsis-induced injury (98). This study established that SIRT3 is a critical node connecting mitochondrial quality control with the core autophagic machinery in GIA. These findings regarding SIRT3-mediated mitochondrial protection have been obtained from murine CLP and endotoxemia models, including intestinal-specific SIRT3 knockout mice; clinical validation in human sepsis cohorts is pending.

Collectively, these findings identify PLK1–mTOR and SIRT3 as parallel regulatory pathways that converge on autophagy and mitochondrial quality control, reinforcing the central role of these processes in maintaining gastrointestinal integrity.

### 4.3 Pharmacologically inducible pathways: teprenone and beyond

The identification of pharmacologically inducible pathways that enhance gastric autophagy further supports its role as a central integration node. Teprenone (geranylgeranylacetone), an anti-ulcer drug with established cytoprotective properties, induces heat shock protein (HSP) expression and protects various tissues under ischemic, hypoxic, and oxidative stress conditions (99–104).

In a rat model of severe burn injury, a clinically relevant model of GIA disruption, teprenone-enriched oral rehydration solution significantly improved 72-h survival (85% vs 45% in untreated animals) and outperformed standard World Health Organization oral rehydration solution (21). Mechanistically, teprenone restored gastric blood flow to near-baseline levels, reduced gastric mucosal injury scores by 63%, and attenuated ileal damage by 58%, revealing that gastric protection propagates along the GIA to preserve distal intestinal integrity (21). These structural improvements were accompanied by significantly reduced oxidative stress and attenuated inflammation, as evidenced by a 47% decrease in malondialdehyde and a 56% decrease in interleukin (IL)-33 levels.

The gastroprotective effects of teprenone are largely attributed to the induction of HSPs (99–104). A systematic drug screening effort further contextualized these findings within the broader landscape of candidate agents for GIA preservation (22). Among 270 eligible studies, 24 candidate drugs were prioritized using a composite bibliometric evidence score that integrated publication frequency, impact metrics, and journal quality. However, experimental validation revealed that only teprenone and vitamin C significantly improved 48-h survival in a burn shock model (80% vs 30% in untreated burns), despite several other agents showing biochemical improvements. This divergence between bibliometric prominence and therapeutic efficacy highlights the importance of mechanism-based screening and supports teprenone as a lead gastroprotective adjunct. It should be noted that the preclinical evidence for teprenone in burn shock models, while promising, derives from a single research group and has not yet been independently replicated. The reported survival benefit therefore requires external validation before it can be considered established evidence for the GIA concept. Independent replication of these findings in other laboratories and trauma models represents a priority for future research.

### 4.4 Additional regulatory mechanisms: heparanase and spermidine

Several other factors have been implicated in autophagy and barrier regulation in GIA. HPA, the only mammalian enzyme capable of degrading heparan sulfate, is upregulated during sepsis and contributes to endothelial dysfunction and organ injury (105, 106). In a rat model of sepsis-induced acute gastrointestinal injury, Chen et al. (107) showed that HPA inhibition with low-molecular-weight heparin significantly improves sepsis-induced acute gastrointestinal injury, reduces serum HPA and syndecan-1 levels, decreases markers of intestinal injury (I-FABP and D-lactate), and improves gastrointestinal motility. Notably, HPA levels are negatively correlated with the autophagy marker LC3B, indicating that HPA inhibition may exert gastroprotective effects by enhancing autophagy (107). The gastroprotective effects of HPA inhibition have been observed in experimental sepsis models; clinical evidence in post-traumatic sepsis patients is not yet available.

Spermidine, a naturally occurring polyamine, improves gut barrier integrity through mechanisms involving autophagy and gut microbiota modulation (108). In murine models, spermidine supplementation enhanced intestinal barrier function, increased TJ protein expression, and reduced permeability. Mechanistically, spermidine activates autophagy in intestinal epithelial cells and alters the gut microbiota composition, increasing the abundance of SCFA-producing bacteria (108). These beneficial effects of spermidine on gut barrier integrity and the microbiome have been demonstrated in murine models of diet-induced obesity; whether similar effects occur in post-traumatic sepsis remains to be investigated.

### 4.5 Cross-talk between autophagy and other cell death pathways

Autophagy interacts dynamically with other programmed cell death pathways, forming a complex regulatory network that determines cell fate in the GIA. Zhang et al. (109) uncovered a critical interaction between necroptotic signaling and the stimulator of the interferon gene (STING) pathway that amplifies inflammation and tissue injury in sepsis. Receptor-interacting protein kinase 3 (RIPK3) and mixed lineage kinase domain-like protein (MLKL), core components of the necroptotic machinery, sustain STING signaling through multiple mechanisms. RIPK3 inhibits autophagic degradation of STING, prolonging its activation, while MLKL exhibits bidirectional regulation. When the pro-necroptotic activity of MLKL is abrogated, MLKL-bound activated STING is secreted extracellularly, limiting the recruitment of TANK-binding kinase 1 and interferon regulatory factor (IRF) 3 (109). These findings revealed that necroptotic signaling proteins have non-canonical roles in modulating inflammatory pathways. Targeting this crosstalk may provide therapeutic benefits in STING-driven inflammatory diseases, including sepsis. This crosstalk between necroptotic signaling and the STING pathway has been characterized in murine sepsis models; its relevance to human post-traumatic sepsis requires further investigation.

Ferroptosis is an iron-dependent form of regulated cell death that has also been recently implicated in sepsis-induced intestinal injury. Hou et al. (110) showed that ghrelin protects against sepsis-induced intestinal injury by inhibiting ferroptosis. In a CLP mouse model, ghrelin reduced bacterial proliferation, attenuated intestinal mucosal damage, decreased systemic inflammation, and preserved mitochondrial integrity. These effects were comparable to those of the ferroptosis inhibitor, ferrostatin-1. Mechanistically, ghrelin modulates ferroptosis markers, reduces lipid peroxidation (4-hydroxynonenal), restores glutathione peroxidase 4 expression, and normalizes iron-handling proteins (ferritin and transferrin) (110). The anti-ferroptotic effects of ghrelin have been demonstrated in a murine CLP model; clinical data in human post-traumatic sepsis are not yet available.

These interconnected pathways—HDAC5–ghrelin–miR-143–ATG2B signaling, PLK1–mTOR-regulated autophagy, SIRT3-mediated mitochondrial protection, and pharmacologically inducible mechanisms such as teprenone—converge on gastric autophagy as a key regulatory node that integrates diverse protective signals to preserve intestinal homeostasis. Until direct evidence from gastric mucosa-specific autophagy models in the context of trauma/sepsis becomes available, the proposed role of gastric autophagy should be viewed as a hypothesis-generating framework rather than a definitively established causal mechanism. Disruption of this node may initiate a cascade that can propagate along the GIA, linking local injury to systemic inflammation and progression to late-onset sepsis.

A structured classification of the evidence supporting each pathway, including model systems, evidence strength, and relevance to post-traumatic sepsis, is provided in **Supplementary Table 2**.

## 5 The GIA: bridging gastric and intestinal dysfunction

The GIA concept integrates gastric and intestinal dysfunctions as interconnected drivers of post-traumatic sepsis. This framework is based on four key principles: (1) gastric injury and impaired autophagy compromise the proximal gastrointestinal barrier, which may lead to the release of damage-associated molecular patterns (DAMPs) and inflammatory mediators; (2) intestinal barrier disruption, dysbiosis, and immune dysfunction amplify and may propagate these inflammatory signals; (3) bidirectional communication occurs through multiple pathways, including lymphatic, humoral, cellular, and neural routes; and (4) targeting the GIA as an integrated system may provide novel therapeutic opportunities.

In this section, we elaborate on these four pillars and provide supporting evidence.

### 5.1 Conceptual framework

The GIA proposes that traumatic injury induces gastric and intestinal hypoperfusion, ischemia-reperfusion injury, and cellular stress. Critically, the stomach response is not passive. Gastric injury is characterized by HDAC5 upregulation, which transcriptionally silences ghrelin expression.

Loss of ghrelin signaling has two major downstream consequences: (1) impaired intestinal barrier function through the miR-143–ATG2B pathway, which leads to intestinal epithelial barrier breakdown; and (2) reduced E2F1 expression, which removes a crucial brake on the NF-κB inflammatory pathway. This leads to the production of a cascade of pro-inflammatory cytokines in the intestinal mucosa.

These inflammatory signals can propagate systemically via multiple routes, including lymphatic, humoral, cellular, and neural pathways. Concurrent intestinal barrier disruption and dysbiosis further amplify and sustain the inflammatory response, establishing a positive feedback loop that can drive distant organ injury, including the lungs, liver, kidneys, muscles, and brain, potentially contributing to chronic critical illness and late-onset sepsis.

### 5.2 Bridging mechanisms

#### 5.2.1 The lymphatic route (gut–lymph hypothesis)

Mesenteric lymphatics constitute the primary conduit for gut-derived inflammatory factors to reach systemic circulation (9–11). The seminal work of Deitch revealed that mesenteric lymph duct ligation prevents hemorrhagic shock-induced lung injury (12) and that post-shock mesenteric lymph, instead of portal plasma, activates neutrophils, increases endothelial permeability, and induces endothelial cell death (13). These effects are mediated by high molecular weight (> 100 kDa) sterile, endotoxin-free factors (111). Although the exact molecular identities of these factors remain incompletely defined, they are distinct from bacteria and endotoxins and likely represent a complex mixture of DAMPs, lipids, and proteases that collectively drive neutrophil activation and endothelial injury. Moreover, the gut lymph pathway contributes to lung injury in both hemorrhagic shock and burn models (112, 113). Gastric lymphatics drain into the mesenteric lymphatic network, providing an anatomical route for gastric-derived factors to reach the intestine.

#### 5.2.2 The humoral route: circulating metabolites, DAMPs, and ghrelin

Circulating factors represent a second major pathway linking gastric and intestinal function. Ghrelin, a gastric-derived hormone whose protective effects on intestinal barrier integrity are mediated through the miR-143–ATG2B autophagy pathway, is a key humoral signal in the GIA (17, 18). In sepsis, HDAC5-mediated suppression of ghrelin production eliminates this protective signal, leaving the intestine vulnerable to injury (19). This loss of hormonal support from the stomach is as critical as the gain of proinflammatory factors from the gut.

In parallel, a range of gut-derived factors enters portal and systemic circulation and influences distant organs. Bacterial outer membrane vesicles, which are spherical, bilayered proteoliposomes containing LPS, proteins, DNA, and RNA, can traverse the intestinal barrier and activate TLR4 and inflammasome pathways, triggering systemic inflammation (114–116). Lcn2, produced by gastric and intestinal epithelial cells, enters the circulation and modulates immune cell function (85). Microbial metabolites, including SCFAs and L-valine, exert both local barrier-protective and systemic anti-inflammatory effects, with many reaching the liver and peripheral organs via the portal circulation (64, 65, 117).

#### 5.2.3 The gut–liver axis

The gut-liver axis represents a critical interface in the pathogenesis of sepsis. Bauer (23) comprehensively reviewed the liver–gut axis as both an initiator and responder to sepsis, emphasizing the essential role of crosstalk between the gut and liver through bacterial translocation and shaping of the microbiome by liver-derived molecules, particularly bile acids. The liver is directly exposed to gut-derived molecules via the portal circulation, while liver-derived molecules, particularly bile acids, shape the intestinal microbiome. In sepsis, bidirectional communication is dysregulated, which contributes to organ failure.

Current clinical assessment of hepatic involvement in sepsis relies primarily on bilirubin within the Sequential Organ Failure Assessment (SOFA) score, which incompletely reflects the liver’s complex metabolic and immunological functions. Advances in sequencing and metabolomics now enable more comprehensive characterization of liver function and its interaction with the microbiome, providing novel opportunities to understand how gut–liver crosstalk and dysbiosis affect susceptibility to and outcomes of sepsis (23). Chen et al. (118) provided an editorial perspective on sepsis-associated liver injury, discussed the mechanisms and potential therapeutic targets, and emphasized that immunosuppression is a key aspect of progressive sepsis.

#### 5.2.4 The cellular route: immune cell trafficking

Immune cells activated by GALT can migrate to distant sites and transmit inflammatory signals. The brain–gut axis in stroke provides a relevant paradigm: Arya and Hu (25) showed that ischemic brain injury induces gut dysbiosis and barrier disruption, promoting migration of gut-derived γδ T cells to the meninges, where they secrete IL-17 and exacerbate neuroinflammation.

A similar mechanism may operate within the GIA. Gastric injury can activate local immune cells, such as macrophages, dendritic cells, and lymphocytes, which may traffic to the intestinal lamina propria via the mesenteric lymphatics or the systemic circulation, propagating inflammation. Conversely, intestinal immune cells may migrate to the stomach and contribute to gastropathy.

Xie et al. (119) provided direct evidence of gut-derived immune cell migration in sepsis-induced acute lung injury (ALI). Using a mouse model of polymicrobial sepsis induced by CLP, the authors revealed that sepsis induces migration of small intestinal memory γδ T17 cells (CD44+ Ly6C- IL-7Rhigh CD8low) to the lung, triggering an IL-17A-dominated inflammatory response. This process was mediated by the activation of wingless-related integration site (Wnt) signaling in alveolar macrophages, which upregulated C-C motif chemokine ligand 1 and facilitated γδ T17 cell migration. Furthermore, esketamine attenuated ALI by inhibiting pulmonary Wnt/β-catenin signaling-mediated migration. These findings highlight the pivotal role of direct gut-to-lung memory T17 cell migration in septic ALI and clarify the importance of localized IL-17A elevation in the lungs.

#### 5.2.5 The neural route: vagus nerve and cholinergic anti-inflammatory pathway

The vagus nerve provides bidirectional communication between the brain and the gastrointestinal tract (120). Efferent vagal fibers activate the cholinergic anti-inflammatory pathway, inhibiting cytokine release from macrophages via α7 nicotinic acetylcholine receptors (121). In parallel, afferent vagal fibers sense luminal contents and transmit signals to the brainstem, thereby modulating gastric and intestinal function (122). In sepsis, vagal dysfunction disrupts this anti-inflammatory pathway, contributing to uncontrolled inflammation (123).

Experimental evidence highlights the importance of this pathway in mediating gut protection. Wu et al. (124) showed that ghrelin ameliorates gut barrier dysfunction in sepsis through vagus nerve activation. In a rat CLP model, intravenous ghrelin reduced serum high-mobility group box 1 levels, decreased intestinal permeability, and attenuated bacterial translocation to mesenteric lymph nodes. These protective effects were completely abolished by vagotomy, revealing that an intact vagus nerve is required for ghrelin action. Intracerebroventricular injection of low-dose ghrelin (1/14th of the systemic dose) recapitulated these protective effects, indicating that ghrelin acts centrally to activate efferent vagal signaling. These findings established the vagus nerve as a critical neural bridge connecting gastric hormone signaling and intestinal protection.

The broader neuroimmune interface further reinforces this concept. Larsson et al. (125) reviewed the innervated gut in critical illness, highlighting brain–gut neuroimmune interactions and their role in the regulation of local and systemic inflammation. The enteric nervous system contains a number of neurons comparable to those in the spinal cord, revealing the capacity for homeostatic reflex regulation of local physiology with neural control of the tissue microenvironment. Emerging therapeutic strategies targeting these neuroimmune pathways have shown promise in preclinical models, indicating that a better understanding of neuroimmune crosstalk in critically ill patients may represent a novel avenue for intervention in sepsis (125).

#### 5.2.6 Prolonged fasting as a clinical amplifier of GIA dysfunction

Prolonged fasting (nil per os, NPO status)—due to hemodynamic instability, repeated surgical interventions, or concerns about feeding intolerance—remains common in ICU practice and may exacerbate GIA dysfunction through several mechanisms. Fasting reduces luminal nutrient sensing, which normally stimulates ghrelin secretion and maintains mucosal integrity. In the absence of luminal nutrients, gastric ghrelin production declines, thereby removing a key trophic signal for intestinal epithelial autophagy. Concurrently, fasting diminishes short-chain fatty acid production by depriving the colonic microbiota of fermentable substrates, compromising colonocyte energy supply and tight junction integrity. Additionally, prolonged NPO status leads to mucosal atrophy, reduced gut-associated lymphoid tissue function, and impaired secretory IgA production, collectively lowering the threshold for bacterial translocation. These fasting-induced changes compound the primary GIA disruption caused by traumatic injury, supporting current guideline recommendations for early enteral nutrition.

#### 5.2.7 Key knowledge gaps in the GIA causal chain

Although the sequential model of gastric dysfunction propagating to intestinal injury is mechanistically plausible and supported by indirect evidence from multiple independent studies, direct causal evidence for this cascade in post-traumatic sepsis remains limited. The finding that teprenone, an agent that primarily acts on the gastric mucosa, reduces distal intestinal injury in a burn model provides the closest available experimental support for gastric-to-intestinal propagation. However, teprenone also induces systemic heat shock protein expression, and its intestinal protective effects may involve both GIA-dependent and GIA-independent mechanisms. Conditional knockout models that selectively disrupt gastric autophagy (e.g., stomach-specific Atg5 or Atg7 deletion) in the context of trauma/sepsis are needed to establish the causal role of gastric autophagy as the initiator of downstream intestinal injury. Until such evidence is available, the GIA model should be viewed as a conceptual framework that generates testable hypotheses rather than a definitively established causal chain.

The heterogeneity of evidence sources and their differential relevance to post-traumatic sepsis are further detailed in **Supplementary Table 2**.

### 5.3 The gut–lung axis: a triple-hit hypothesis

Ziaka and Exadaktylos (126) proposed a “triple-hit hypothesis” to explain the pathophysiology of ALI in patients with acute brain injury, which integrates the emerging concept of the gut–lung axis. In this framework, the initial brain injury triggers sympathetic hyperactivity, a well-recognized contributor to ALI. Concurrently, the direct effects of acute brain injury, including inflammation and oxidative stress, constitute the “first hit,” rendering the lungs vulnerable to additional insults. Subsequent interventions, such as mechanical ventilation, constitute the “second hit,” further exacerbating lung vulnerability. Finally, the “third hit” arises from dysbiosis and intestinal dysfunction in patients with acute brain injury, initiating a cascade of events involving immune dysregulation and microbiome alterations, which subsequently affect the lungs. Collectively, these processes amplify pulmonary inflammation and contribute to ALI development or progression (126).

Therapeutic targeting of this axis has been explored in experimental models. Peng et al. (127) developed a novel therapeutic approach targeting this axis using oral multi-enzymatic manganese-carbon dots (Mn-CDs), which exhibit potent reactive oxygen species-scavenging activity. In murine sepsis models, Mn-CD treatment improved systemic indices and promoted macrophage efferocytosis. Mechanistically, Mn-CDs enrich beneficial gut bacteria (*Clostridium* and *Bacteroides*) and increase indole-3-propionic acid production, which activates AHR to promote anti-inflammatory macrophage polarization. Fecal microbiota transplantation from Mn-CD-treated mice recapitulated these protective effects, whereas microbiota depletion abolished them, highlighting the essential role of gut microbiota in mediating this gut–lung protective axis (127).

Wei et al. (128) revealed improved survival and mitigated lung injury through the augmentation of CD4+Foxp3+ regulatory T cells and enhancement of gut and lung barrier function in CLP mice administered sodium butyrate gavage. These findings further support the therapeutic potential of targeting the gut–lung axis in sepsis.

### 5.4 Other emerging axes

Beyond the gut–liver and gut–lung axes discussed above, additional organ–gut axes—including the gut–temperature, gut–muscle, gut–kidney, and gut–heart axes—have been increasingly recognized in sepsis pathogenesis. A recent comprehensive review by Wang et al. (129) consolidated the bidirectional interplay between gut dysbiosis and multi-organ dysfunction across these axes and underscored the therapeutic potential of microbiota-based interventions in this context. A summary of these additional axes is provided in **Supplementary Table 4**.

## 6 Biomarkers of gastrointestinal dysfunction: from structural damage to functional impairment

Clinical assessment of gastrointestinal dysfunction in critically ill patients remains limited, relying largely on nonspecific signs such as abdominal distension, diarrhea, and feeding intolerance. The Acute Gastrointestinal Injury (AGI) grading system, proposed by the European Society of Intensive Care Medicine in 2012, provides a standardized framework for assessing the severity of gastrointestinal dysfunction based on clinical symptoms, intra-abdominal pressure, and feeding intolerance (130).

Hu et al. (131) validated this system in a multicenter prospective study of 550 critically ill patients, revealing that global AGI grade was independently associated with 60-day mortality (hazard ratio 1.65; 95% confidence interval 1.28–2.12) and that persistent feeding intolerance within the first week of ICU stay provided incremental prognostic value. However, reliance on clinical assessment alone is insufficient, and reliable biomarkers are urgently needed to identify at-risk patients, monitor disease progression, and guide therapeutic interventions (132).

### 6.1 Established biomarkers of intestinal injury

I-FABP is a cytosolic protein expressed in enterocytes and released upon cell necrosis (133, 134). Elevated plasma or urinary I-FABP levels are correlated with intestinal ischemia, sepsis severity, and mortality (135–138). A meta-analysis reported a pooled sensitivity of 80% and a specificity of 85% for the diagnosis of acute intestinal ischemia (139). In patients with trauma, I-FABP levels peak immediately after injury and are higher in non-survivors, which is correlated with hemodynamic compromise (140, 141). In Russell’s viper envenomation, I-FABP elevation occurs independent of abdominal pain, indicating that symptoms are unreliable surrogates for enterocyte damage (142). In sepsis, I-FABP levels are elevated early and can predict mortality, particularly in patients with septic shock (143, 144).

D-lactate, produced almost exclusively by bacterial fermentation, serves as a marker of intestinal barrier dysfunction when it enters the systemic circulation (145, 146). Elevated levels have been reported in mesenteric ischemia and sepsis, and following major surgeries (147, 148). Li et al. (149) showed that lactate levels increase progressively with AGI grade and independently predict AGI severity, outperforming I-FABP and LPS. In another study, D-lactate was superior to fecal calprotectin and IL-6 in predicting AGI in patients with sepsis (150).

Citrulline, a non-protein amino acid synthesized by enterocytes, reflects functional enterocyte mass (151, 152). Low citrulline levels are associated with intestinal failure, sepsis severity, and mortality (153, 154), and independently predict subsequent GI failure in patients with sepsis (143). However, this interpretation is complicated by renal function and postprandial variations (155).

Zonulin is the only known physiological modulator of intercellular TJs, and serves as a marker of barrier permeability (156). Elevated plasma zonulin levels indicate TJ dysfunction and have been reported in patients with sepsis. These levels are also correlated with disease severity (157, 158).

Members of the trefoil factor family, particularly TFF2 and TFF3, are secreted by mucosal cells and are upregulated after injury to promote epithelial restitution (159, 160). TFF2 predicts mortality in children with multiple organ dysfunction syndrome (MODS) (159), while TFF3 is correlated with organ failure in abdominal sepsis but does not independently predict outcomes (160).

### 6.2 The need for functional biomarkers

While established biomarkers reflect structural damage, barrier dysfunction, and enterocyte mass, they do not capture the functional capacity of the gastric hormone axis. Circulating ghrelin has been proposed as a potential functional biomarker of GIA integrity, reflecting the capacity of the gastric endocrine axis to maintain downstream intestinal protection (94, 96). However, the clinical application of ghrelin measurement faces significant challenges, including the lack of standardized clinical-grade assays, undefined reference ranges in critically ill patients, and substantial confounding by nutritional state and circadian rhythm. Given these challenges, ghrelin measurement should be viewed as an investigational approach requiring substantial development before clinical implementation.

### 6.3 Microbiome signatures as prognostic biomarkers

The composition of the gut microbiota has emerged as a powerful prognostic tool. Progressive *Enterobacteriaceae* enrichment in critically ill patients is associated with a 6.8-fold increased odds of nosocomial infection (56). Similarly, intestinal domination by *Enterococcus* (≥ 30% relative abundance) at ICU admission is linked to increased risk of death (162), with each log-unit increase in *Enterococcus* abundance associated with a more than threefold increase in ICU mortality (163).

Fecal metabolite profiles also have prognostic value. Sustained reduction in SCFAs, particularly butyrate and propionate, is associated with increased mortality (66). Additionally, in patients with severe burns, undetectable SCFA levels are associated with fatal outcomes (164).

Integration of microbiome sequencing with metabolomic profiling provides a multi-dimensional approach to risk stratification, enabling identification of patients with high-risk dysbiosis who may benefit from early microbiota-directed interventions.

### 6.4 Integrating biomarkers with AGI grading: a multi-modal approach

Optimal assessment of gastrointestinal dysfunction requires integration of multiple modalities. Incorporating AGI grade on day 1 alongside clinical risk factors significantly improves mortality prediction (131). Similarly, combining D-lactate with Acute Physiology and Chronic Health Evaluation II and intra-abdominal pressure improves discrimination of AGI severity (149).

**Figure 3:**
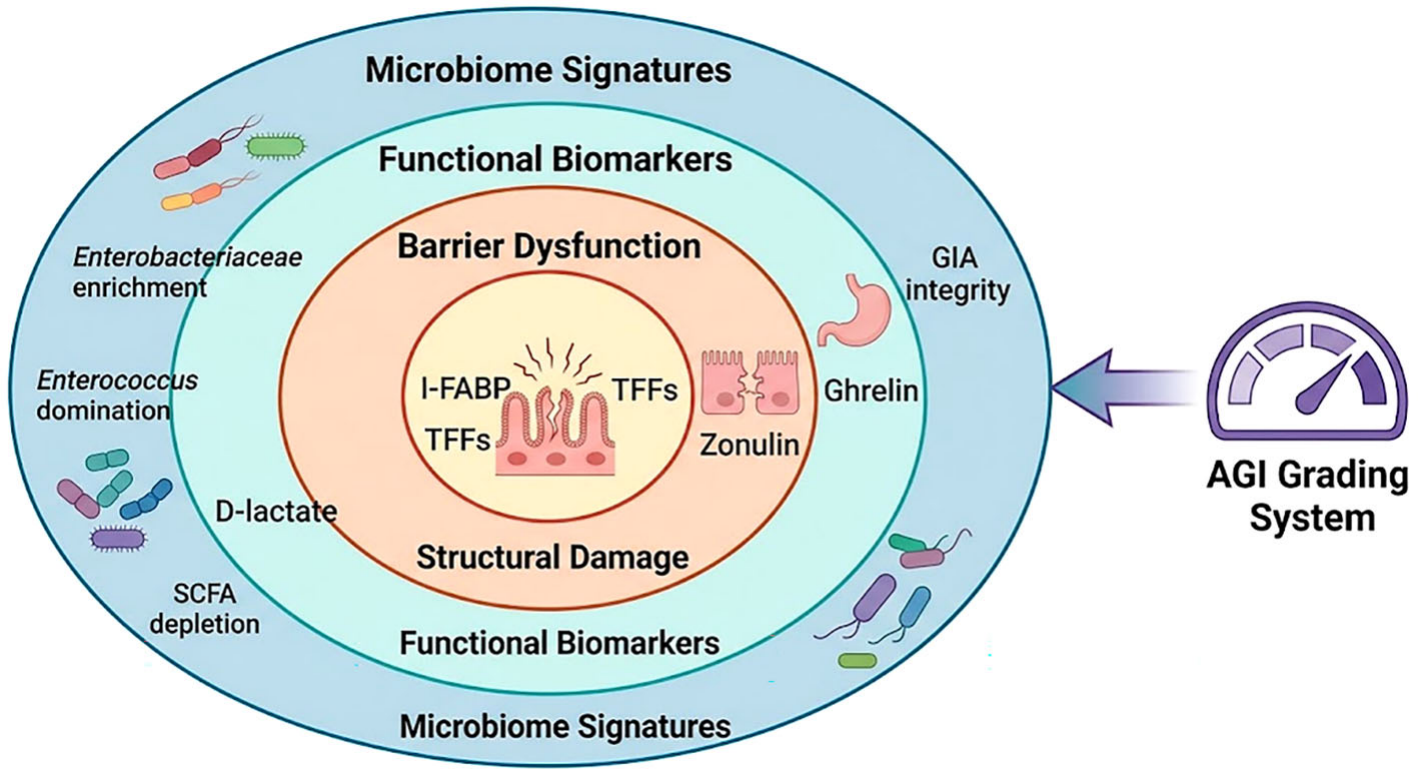
Multi-layered biomarkers for GIA assessment. Biomarkers reflecting different aspects of gastrointestinal dysfunction can be integrated to enable comprehensive GIA assessment. Structural damage markers (intestinal fatty acid-binding protein [I-FABP], zonulin, trefoil factor family [TFF] proteins) indicate enterocyte injury and barrier disruption. Functional markers (citrulline, D-lactate) reflect enterocyte mass and barrier permeability. Regulatory markers (ghrelin) capture the functional status of the gastric endocrine axis. Microbiome signatures (Enterobacteriaceae enrichment, short-chain fatty acid [SCFA] depletion) provide prognostic information regarding dysbiosis severity. Integration of these biomarkers with clinical grading (Acute Gastrointestinal Injury [AGI] grade) enables multimodal risk stratification. AGI, Acute Gastrointestinal Injury; GIA, Gastrointestinal Axis; I-FABP, intestinal fatty acid-binding protein; SCFA, short-chain fatty acid; TFF, trefoil factor family. .

These findings support a multimodal approach that integrates clinical grading, circulating biomarkers, and imaging to enable comprehensive assessment of GI function (150). Such an approach may inform the development of personalized therapeutic strategies targeting the GIA (**Figure 3**).

Several general limitations warrant consideration when interpreting gastrointestinal biomarkers in the ICU setting. First, renal function is a significant confounder for several markers, as both citrulline and D-lactate are partially cleared by the kidneys; acute kidney injury, which is common in post-traumatic sepsis, may elevate these markers independently of gastrointestinal injury (154). Second, many biomarkers lack standardized cut-off values for critically ill populations, with most reference ranges derived from healthy volunteers or non-septic patients. Third, nutritional status and feeding route profoundly influence several markers—citrulline levels depend on glutamine availability, while SCFA production requires enteral delivery of fermentable substrates. Fourth, antibiotic exposure is a major confounder for microbiome-derived markers, as broad-spectrum antibiotics rapidly and profoundly alter gut microbial composition and metabolite production (56, 57). Finally, the feasibility of real-time biomarker-guided decision-making remains limited: most assays require specialized laboratory equipment with turnaround times of hours to days, and point-of-care testing for gastrointestinal biomarkers is largely unavailable. These limitations are systematically summarized in **Table 1**.

**Table 1:** Critical appraisal of gastrointestinal biomarkers in post-traumatic sepsis.

Biomarker	Specificity for GI injury	Assay standardization	Key confounders	Optimal sampling timing	Real-time ICU feasibility
I-FABP	High for enterocyte necrosis; also elevated in mesenteric ischemia, abdominal trauma	ELISA available; lack of standardized cut-off values across studies; inter-assay variability	Renal function (partially renally cleared); timing after injury critical	Peak within hours of injury; serial measurements more informative than single values	Moderate—ELISA turnaround ~2–4 h; point-of-care assays in development
D-lactate	Moderate—produced by bacterial fermentation; reflects barrier permeability rather than direct injury	Enzymatic assays available; lack of age- and comorbidity-adjusted reference ranges	Renal function (renally cleared); bacterial overgrowth; probiotic use; dietary intake	Elevation may lag behind injury onset; sustained elevation suggests ongoing barrier dysfunction	High—enzymatic assays routinely available in clinical labs
Citrulline	High for functional enterocyte mass; correlates with absorptive capacity	HPLC or enzymatic assays; reference ranges established for healthy populations but not for critically ill	Renal function strongly influences levels; postprandial variations; nutritional support (especially parenteral nutrition)	Declines progressively with enterocyte loss; not suitable for acute detection	Moderate—HPLC not routinely available; enzymatic assays more feasible
Zonulin	Moderate—modulates TJ permeability; elevated in multiple conditions (sepsis, autoimmune disease, obesity)	ELISA available; wide variation in reported normal ranges; lack of standardized preanalytical protocols	Systemic inflammation (non-specific elevation); metabolic syndrome; autoimmune conditions	Unclear; limited data on kinetics in acute illness	Low—ELISA turnaround time limits real-time use
TFF2/3	Moderate—mucosal restitution markers; TFF2 elevated in MODS; TFF3 correlates with organ failure	ELISA available; limited clinical validation in sepsis cohorts	Multiple organ dysfunction (TFF2); non-GI sources of inflammation	TFF2 peaks early in MODS; TFF3 kinetics not well characterized	Low—research use only; limited clinical availability
Ghrelin	Potentially high for GIA functional status, but unproven; reflects gastric endocrine function rather than injury	No standardized clinical assay; research-grade ELISA/RIA only; acyl vs. des-acyl ghrelin distinction critical	Nutritional status (fed/fasted state strongly influences levels); circadian rhythm; renal function; age; body composition	Fasting morning samples preferred; serial measurements needed to assess axis function	Very low—specialized sample handling required; no point-of-care option
Microbiome signatures	High for dysbiosis patterns; Enterobacteriaceae enrichment strongly associated with nosocomial infection risk	16S rRNA sequencing or metagenomics; no standardized clinical protocols; bioinformatics pipelines vary between studies	Antibiotic exposure (major confounder); nutritional route (enteral vs. parenteral); PPI use; sample type (rectal swab vs. stool)	Changes occur within hours of insult; longitudinal sampling more informative than single timepoints	Very low—turnaround time days to weeks; research setting only

ELISA, enzyme-linked immunosorbent assay; GC-MS, gas chromatography-mass spectrometry; GI, gastrointestinal; GIA, Gastrointestinal Axis; HPLC, high-performance liquid chromatography; I-FABP, intestinal fatty acid-binding protein; ICU, intensive care unit; MODS, multiple organ dysfunction syndrome; PPI, proton pump inhibitor; RIA, radioimmunoassay; SCFA, short-chain fatty acid; TFF, trefoil factor family; TJ, tight junction.

## 7 Therapeutic strategies targeting the GIA

Recognition of gastric and intestinal dysfunctions as interconnected drivers of post-traumatic sepsis opens new avenues for therapeutic intervention. Strategies may target individual components of the GIA or the integrated axis as a whole. A systematic ranking of the evidence supporting each therapeutic strategy, including source of evidence, availability of human data, and translational readiness, is provided in **Supplementary Table 3**.

### 7.1 Conceptual foundation: from bacterial translocation to the gut–lymph hypothesis

The gut-origin hypothesis of sepsis was initially based on the concept that failure of the gut barrier permits translocation of derived bacteria and endotoxins into the bloodstream and systemic tissues to trigger sepsis and promote MODS (165). Epstein et al. (166) defined the “motor of sepsis” concept, which emphasizes the critical role of the gastrointestinal barrier, comprising mechanical, immunologic, and microbial components, in maintaining host homeostasis. Under stress conditions, including thermal injury, trauma, surgery, and malnutrition, immunosuppression, this barrier is compromised. Burn wounds are frequently colonized by gastrointestinal tract flora, and the passage of inert particles and microorganisms across the intestinal wall, termed bacterial translocation, has been proposed as the initiating event (6–8).

However, subsequent human studies challenge this framework. Moore’s portal vein catheterization study in trauma patients failed to detect significant bacteremia or endotoxemia, indicating that translocating bacteria and endotoxins alone may not be primarily or exclusively responsible for gut-induced MODS (167, 168). This apparent paradox led to the gut–lymph hypothesis, which proposes that gut-derived pro-inflammatory factors are transported via the mesenteric lymphatics instead of the portal circulation (9–13). Deitch’s seminal experimental work showed that mesenteric lymph duct ligation prevents hemorrhagic shock-induced lung injury, and that post-shock mesenteric lymph, unlike portal plasma, activates neutrophils, increases endothelial permeability, and induces endothelial cell death (12, 13). These findings establish the ischemic gut as a source of pro-inflammatory mediators capable of driving distant organ and cellular dysfunctions (9, 12).

This conceptual evolution has profound therapeutic implications. Effective interventions targeting the GIA must consider not only the intestinal lumen and mucosa but also the mesenteric lymphatics as a critical conduit for gut-derived inflammatory mediators.

### 7.2 Established gut-directed therapies

#### 7.2.1 Early enteral nutrition

Early enteral nutrition (EEN), defined as initiation within 24–48 h of injury or ICU admission, provides strong clinical and experimental support for gut-directed therapy. The rationale for EEN is based on experimental studies showing that enteral feeding preserves the structural and functional integrity of the gut. In a guinea pig burn model, Mochizuki et al. (169) showed that continuous enteral feeding starting at 2 h post-burn significantly decreased the metabolic rate at 2 weeks. Inoue et al. (170) reported that a single bolus of tube feeding 12 h after thermal injury markedly decreases *Candida albicans* translocation. These effects are attributed to the preservation of gut blood flow (169), maintenance of mucosal mass (171), stimulation of epithelial cell proliferation (170), and production of trophic endogenous mediators that collectively support gut integrity (166, 171, 172).

Clinical studies have corroborated these findings. A meta-analysis by Pu et al. (173) of seven randomized controlled trials (RCTs) including 527 patients with major burns revealed that EEN initiated within 24 h significantly reduced mortality (OR 0.36; 95% CI 0.18-0.72), gastrointestinal hemorrhage, sepsis, pneumonia, renal failure, and hospital length of stay. A more recent systematic review by Grillo-Ardila et al. (174) in patients with sepsis or septic shock found low-certainty evidence that EEN reduces the number of days of mechanical ventilation and lowers SOFA scores, although it is associated with increased diarrhea.

The 2019 European Society for Clinical Nutrition and Metabolism (ESPEN) guidelines provide comprehensive recommendations (175): early EN (within 48 h) should be initiated instead of delayed; early EN is preferred over early parenteral nutrition (PN); and nutrition should be prescribed progressively to avoid overfeeding.

Mechanistic insights from Sigalet et al. (176) explain why EEN is superior to PN. Animals maintained on total parenteral nutrition (TPN) show significant alterations in GALT, including decreased CD4:CD8 ratio and IL-4, IL-10, and sIgA levels, which compromise immunity (177–179). These changes increase susceptibility to infectious challenges (180). The enteral administration of a complex diet reversed these abnormalities. Supplementation of TPN with 2% glutamine maintains normal Peyer’s patch cell populations and Th2 cytokine production (181), while the enteric neuropeptide bombesin preserves GALT integrity and mucosal immunity (172).

#### 7.2.2 Glutamine

GLN is the most abundant free amino acid and is a critical fuel for enterocytes, lymphocytes, and macrophages (182–185). During catabolic states, GLN becomes conditionally essential, with plasma levels declining dramatically (186, 187), a change linked to impaired immune function and increased mortality (188, 189).

The metabolic rationale for GLN supplementation is multifactorial (190). GLN supports cellular energy metabolism, nucleotide synthesis, antioxidant defense, HSP induction, immune modulation, and gut barrier function. Mechanistic studies have uncovered novel pathways by which GLN exerts protective effects in burn sepsis, including the preservation of liver mitochondrial function (191) and maintenance of intestinal mucus barrier integrity (192).

Clinical evidence, however, has been inconsistent. Early meta-analyses revealed potential benefits of GLN supplementation (193, 194), but these findings were challenged by two large RCTs. The REducing Deaths due to Oxidative Stress trial reported increased 28-day mortality in critically ill patients receiving GLN (195), while the RE-Evaluating the Efficacy of ENteral ERGlutamine in Thermal Injury trial in patients with burns found no significant improvements in mortality or infectious complications with enteral GLN (196). A recent meta-analysis confirmed that while GLN shortens hospital stay and improves wound healing, it does not improve in-hospital mortality (197).

Accordingly, the 2019 ESPEN guidelines recommend a nuanced approach (175). Enteral GLN may be considered in patients with severe burns (> 20% total body surface area) but routine use in unselected critically ill populations, particularly those with organ failure, is not recommended (198, 199). It should be noted that the mechanistic findings regarding mitochondrial preservation and mucus barrier maintenance described above are derived from animal models and have not yet been validated in human cohorts with post-traumatic sepsis.

#### 7.2.3 Nutritional route: enteral vs parenteral

The superiority of enteral over parenteral nutrition is firmly established. PN has largely been replaced by EN because of the increased risk of infectious complications, liver dysfunction, and mortality with PN (200, 201). Herndon et al. (202) reported increased mortality for patients administered supplemental PN than for those administered EN alone in patients with severe burns. PN also promotes the secretion of proinflammatory mediators and aggravates fatty infiltration in the liver (203, 204).

The advantages of EN include the preservation of gut mucosal architecture and function (169, 171), stimulation of splanchnic blood flow (169), decreased bacterial translocation (6–8), maintenance of GALT and sIgA production (178, 179), blunted stress hormone response (205), lower cost, and fewer line-related infections (206). The ESPEN guidelines recommend EN initiation within 48 h (175).

### 7.3 Microbiota-directed therapies

Recognition that gut microbiota dysbiosis is both a consequence and a driver of critical illness has spurred interest in therapies targeting the microbiome (49, 58, 59).

#### 7.3.1 Probiotics

Probiotics are defined by the WHO as “live microorganisms which, when administered in adequate amounts, confer a health benefit on the host.” (207) The proposed mechanisms of these benefits include competitive exclusion of pathogens (208), production of antimicrobial substances (209), enhancement of intestinal barrier function (210), immunomodulation (211, 212), and SCFA production.

Meta-analyses have revealed the benefits in specific populations. Gu et al. (213) found that probiotics reduced nosocomial infections and VAP in patients. Du et al. (214) reported reduced infection and mortality rates in patients with severe craniocerebral injury. Wang et al. (215) observed a significant reduction in VAP with probiotics in critically ill adults, with synbiotics being more effective than probiotics alone.

However, evidence from large randomized trials remains inconclusive. The largest and most rigorous trial to date, the Probiotics: Prevention of Severe Pneumonia and Endotracheal Colonization Trial, which randomized 2,650 mechanically ventilated patients to *L. rhamnosus* GG or placebo, found no difference in VAP, *Clostridioides difficile* infection, or mortality between groups, but noted an increased incidence of serious adverse events (216). Similarly, a comprehensive meta-analysis by Sharif et al. (217) concluded that while probiotics may confer benefits, they are also associated with increased risks of adverse events, including the isolation of probiotic organisms from sterile sites.

Safety concerns have been well-documented. The Probiotics in Pancreatitis Trial for severe acute pancreatitis showed increased mortality and bowel ischemia with a multispecies probiotic (218). Case reports of *Lactobacillus* and *S. boulardii* bacteremia among patients in the ICU have been linked to central venous catheter contamination (219). Yelin et al. (220) provided genomic evidence of bacterial transmission from probiotic capsules to blood.

Importantly, the risk–benefit profile of probiotics in post-traumatic sepsis and critical illness warrants particular scrutiny. Patients with post-traumatic sepsis exhibit impaired intestinal barrier integrity, which may facilitate the translocation of administered live microorganisms across the gut epithelium into the systemic circulation. Furthermore, immune dysregulation—characterized by both hyperinflammation and compensatory immunosuppression—may impair the host’s ability to contain translocated microbial strains. The concomitant use of central venous catheters, which is nearly universal in ICU care, provides an additional route through which probiotic organisms may enter the bloodstream, as evidenced by genomic documentation of probiotic-to-blood transmission (220). Given these considerations, although probiotics may offer benefits in selected patient populations, their routine use in post-traumatic sepsis cannot be recommended without careful individualized risk–benefit assessment. The administration of live microorganisms to patients with compromised gut barrier function should be considered an intervention with genuine potential for harm, and probiotic use in this context remains experimental pending further safety data from adequately powered randomized controlled trials in trauma and burn populations.

#### 7.3.2 Synbiotics

Synbiotics combine probiotics and prebiotics to enhance microbial viability and function. In a RCT including 72 mechanically ventilated patients with sepsis, Shimizu et al. (221) reported that synbiotics significantly reduced enteritis and VAP and increased beneficial bacteria and fecal organic acids, with no differences in bacteremia or mortality. Meta-analyses have also confirmed the superiority of synbiotics over probiotics alone in reducing infectious complications (215, 222). However, as synbiotics contain live microorganisms, they share the same theoretical safety concerns as probiotics, particularly regarding the risk of microbial translocation in patients with compromised intestinal barrier function. Safety data for synbiotics specifically in post-traumatic or burn populations remain limited, and their use in these patients should be approached with similar caution.

#### 7.3.3 Postbiotics

Postbiotics, defined as preparations of inactivated microorganisms or their components, offer potential advantages over live probiotics, including improved safety and stability (223). SCFAs are the most well-characterized postbiotics. In sepsis, fecal SCFA levels decline and correlate with disease severity and mortality, whereas supplementation improves survival in experimental models and confers protection against sepsis-associated organ dysfunction, including encephalopathy via G protein–coupled receptor signaling (66–69, 224).

A recent comprehensive review by Benvenuto et al. (225) further consolidated preclinical evidence for butyrate in sepsis, showing improved survival (20%–40%) in animal models and highlighting its multi-organ protective effects (neurologic, hepatic, intestinal, cardiac, pulmonary, and renal). Other postbiotics, such as bacteriocins and exopolysaccharides, have shown antimicrobial and barrier-protective effects in preclinical studies (226–232); however, clinical evidence remains limited. A recent comprehensive review by Xu (233) elucidated the direct immune targets of gut microbiota metabolites in sepsis, highlighting the potential for integrating artificial intelligence (AI) with microbiota-based therapies.

#### 7.3.4 FMT

FMT is the most radical approach to microbiota restoration. A recent analysis of the global clinical trial landscape identified FMT as one of the most extensively studied microbiome modulators in sepsis, with 12% of registered trials focusing on this approach (234). Preclinical studies demonstrated that FMT rescues mice from lethal polymicrobial sepsis by restoring IRF3-dependent systemic immunity (235, 236). Although case reports reveal a potential benefit in patients with sepsis with refractory diarrhea (237), FMT carries risks, including the transmission of drug-resistant pathogens, donor-derived infections, and the theoretical risk of transferring a pathogenic microbiome to an immunocompromised host. In post-traumatic sepsis patients—who exhibit both intestinal barrier disruption and immune dysregulation—these risks are heightened, as the same barrier defects that permit translocation of endogenous pathobionts may also facilitate systemic dissemination of donor microorganisms. Consequently, its use remains experimental in critical illnesses and should be restricted to clinical trials in post-traumatic sepsis patients. Future directions include development of defined microbial consortia and targeted modulation of microbiota-driven organ axes, such as the gut–lung axis (238).

### 7.4 Novel mechanism-based therapies

#### 7.4.1 Ghrelin axis restoration

Identification of the HDAC5–ghrelin–E2F1–NF-κB axis opens multiple therapeutic avenues (19). HDAC5 inhibitors, such as LMK-235, restore endogenous ghrelin production, enhance autophagy, and suppress inflammation (18, 19). In preclinical studies, direct ghrelin administration protects against sepsis-induced intestinal injury, lung injury, and mortality (17, 124) by enhancing autophagy (18), M2 macrophage polarization (112), and vagus nerve activation (124). Although the preclinical evidence supporting ghrelin axis restoration is compelling, these strategies have not yet been evaluated in human post-traumatic sepsis cohorts, and their clinical translation awaits further pharmacokinetic, safety, and dosing studies.

#### 7.4.2 Other promising preclinical targets

In addition to the ghrelin axis, several other pharmacological targets have been identified. For instance, PLK1 regulates intestinal epithelial autophagy through mTOR inhibition; although direct activators are not yet available, mTOR inhibitors such as rapamycin may indirectly enhance PLK1 expression (97). Melatonin activates SIRT3 and protects against sepsis-induced intestinal injury (114). HPA inhibition using low-molecular-weight heparin enhances autophagy and protects glycocalyx integrity (107). The natural polyamine spermidine improves gut barrier integrity through combined effects on autophagy and the gut microbiota (108).

Teprenone, an anti-ulcer agent, induces HSP expression in the gastric mucosa (239) and improves survival and gastric blood flow in burn shock models (21, 22).

These findings remain at the preclinical stage, and corresponding human data are not yet available.

#### 7.4.3 Emerging approaches targeting the microbiota–host interface

**Figure 4:**
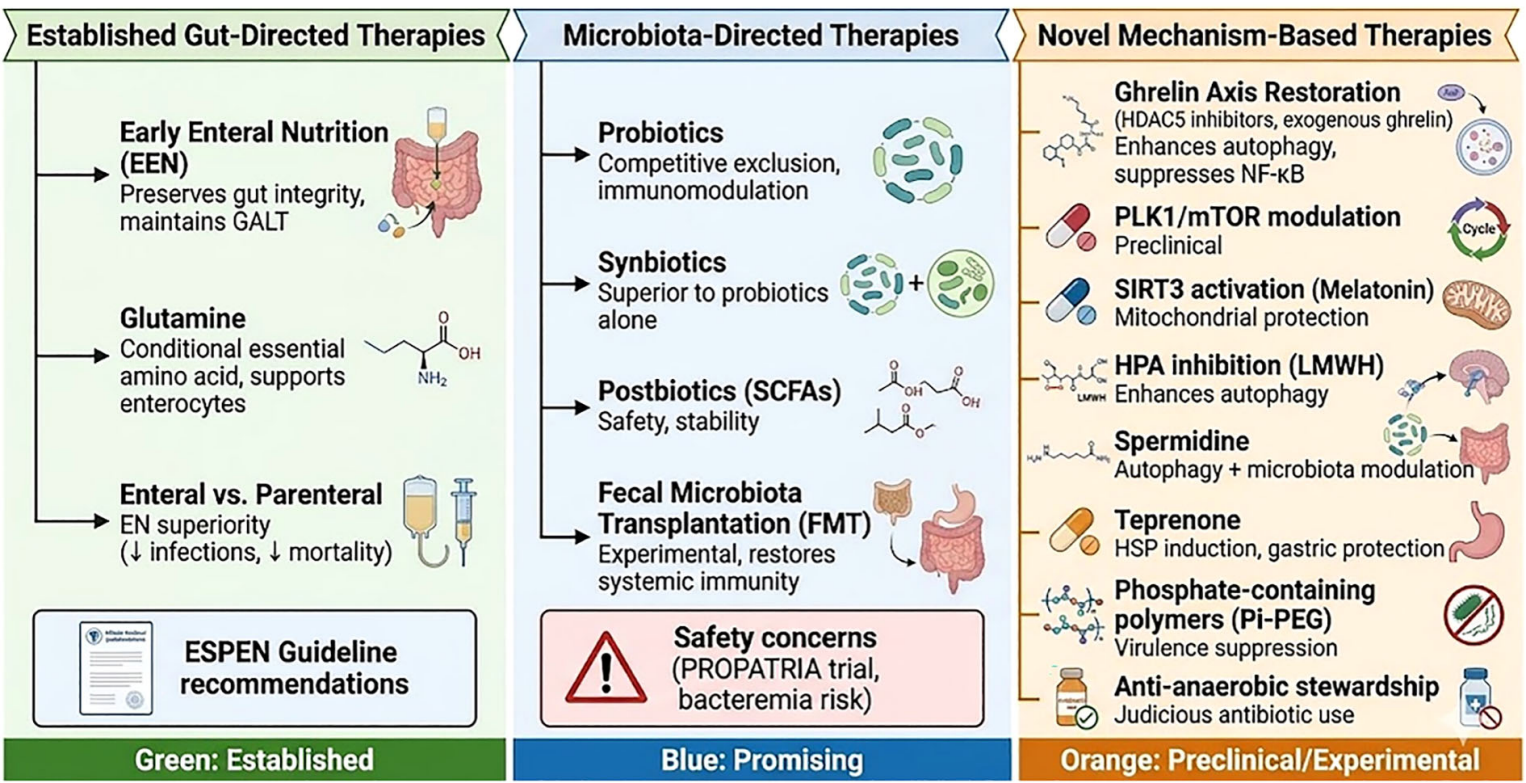
Therapeutic strategies targeting the GIA. Interventions are organized by their level of clinical evidence and mechanistic rationale. Established therapies include early enteral nutrition (recommended by ESPEN guidelines within 48 hours) and selective glutamine supplementation in severe burns. Microbiota-directed strategies encompass probiotics, synbiotics (combining probiotics with prebiotics), postbiotics such as short-chain fatty acids (SCFAs), and fecal microbiota transplantation (FMT)—all requiring careful safety consideration in critically ill patients. Mechanism-based approaches targeting specific GIA nodes include: HDAC5 inhibition (LMK-235) to restore ghrelin–E2F1–NF-κB signaling; PLK1 activation or mTOR inhibition to enhance autophagy; SIRT3 activation (e.g., via melatonin) to preserve mitochondrial function; heparanase (HPA) inhibition with low-molecular-weight heparin (LMWH) to protect glycocalyx integrity; and teprenone to induce cytoprotective heat shock proteins. Phosphate-containing polymers (Pi-PEG) represent an emerging strategy to suppress pathobiont virulence at the microbiota–host interface. E2F1, E2F transcription factor 1; ESPEN, European Society for Clinical Nutrition and Metabolism; FMT, fecal microbiota transplantation; GIA, Gastrointestinal Axis; HDAC5, histone deacetylase 5; HPA, heparanase; LMWH, low-molecular-weight heparin; mTOR, mechanistic target of rapamycin; NF-κB, nuclear factor kappa-B; Pi-PEG, phosphorylated polyethylene glycol; PLK1, polo-like kinase 1; SCFAs, short-chain fatty acids; SIRT3, sirtuin 3.

Additional strategies target the interface between the gut microbiota and host immunity. Phosphate-containing polymers, such as phosphorylated polyethylene glycol, suppress pathobiont virulence by sequestering phosphate, reducing lethality in sepsis models (240–242). Anti-anaerobic antibiotic stewardship is also critical: anti-anaerobic antibiotics increase VAP and mortality in mechanically ventilated patients by depleting anaerobes and promoting pathogen expansion, thus supporting more judicious antibiotic use (59) (**Figure 4**).

## 8 Discussion

### 8.1 From concept to clinical practice

Translating the refined GIA concept into clinical practice requires a systematic, stepwise approach. First, the molecular pathways identified in preclinical models, particularly the HDAC5–ghrelin–E2F1–NF-κB axis, the PLK1–mTOR pathway, and SIRT3-mediated mitochondrial protection, must be validated in human sepsis cohorts. Prospective observational studies measuring serial biomarker levels and correlating them with clinical outcomes will be essential to establish clinical relevance. Concurrently, the development of rapid point-of-care assays for key GIA biomarkers, including ghrelin, I-FABP, D-lactate, and citrulline, is urgently required to enable real-time risk stratification and guide therapeutic decision-making. Integration of biomarker data with clinical AGI grading, as reported by Hu et al. (131) and Li et al. (149), provides a multimodal approach that may enhance prognostic accuracy.

Once validated biomarkers and assays are available, clinical trials of GIA-targeted interventions, particularly HDAC5 inhibitors, ghrelin supplementation, melatonin, and teprenone, should incorporate stratification based on baseline biomarker profiles, such as low ghrelin levels or *Enterobacteriaceae* enrichment, to maximize the likelihood of detecting therapeutic benefits. The inherent heterogeneity of sepsis, driven in part by differences in gut microbiome composition and immune responses, highlights the need for personalized treatment strategies (243). Careful safety monitoring is essential, particularly for HDAC inhibitors, which may have off-target epigenetic effects, and for any intervention involving live microorganisms in patients with compromised gut barrier function. A recent comprehensive analysis of 112 clinical trials highlights the growing global commitment to microbiome-based therapies for sepsis, with the Asia–Pacific region emerging as a rapidly growing research hub (234). Finally, given evidence of sex-related differences in post-injury dysbiosis (58, 59), clinical trials should be designed to enable sex-stratified analyses and assess differential treatment effects between male and female patients.

### 8.2 Limitations of the proposed GIA model

A further limitation of the current evidence base is the heterogeneity of experimental models from which GIA-supporting data are derived. As detailed in **Supplementary Table 2**, the evidence assembled in this review spans trauma- and burn-specific models (e.g., burn shock with teprenone, hemorrhagic shock with mesenteric lymph), general sepsis models (e.g., CLP, LPS endotoxemia), and non-sepsis contexts (e.g., diet-induced obesity for spermidine). These models differ substantially in their fidelity to human post-traumatic sepsis pathophysiology. In particular, CLP and LPS models, while widely used in sepsis research, do not recapitulate the tissue hypoperfusion and ischemia–reperfusion injury that characterize the early post-traumatic period and are central to the proposed GIA cascade. This heterogeneity reflects the current state of the field, in which trauma-specific gastrointestinal research remains limited relative to general sepsis research. Key GIA pathways that currently lack validation in trauma- and burn-specific models—including the PLK1–mTOR axis, SIRT3-mediated mitochondrial protection, and ghrelin’s anti-ferroptotic effects—represent priority areas for future investigation. The translational readiness of key GIA pathways and interventions, classified into four tiers from guideline-supported to emerging, is systematically presented in **Supplementary Table 3**. Together, **Supplementary Tables 2**, **3** provide a comprehensive evidence map: S2 classifies evidence by the model system from which it was derived and its relevance to post-traumatic sepsis, while S3 ranks the same evidence by translational readiness for clinical application.

Several specific knowledge gaps warrant emphasis. First, direct evidence for gastric mucosal autophagy in human post-traumatic sepsis remains absent; the current mechanistic framework is based primarily on data from intestinal epithelial cells, CLP/LPS models, and ghrelin-mediated signaling, while direct assessment of autophagic flux in the gastric mucosa of patients with trauma has not been performed. Second, the clinical utility of the ghrelin axis as a functional GIA biomarker is constrained by the lack of standardized assays, demanding sample handling requirements, and the absence of established reference ranges in critically ill populations. Third, the comparability of findings across different trauma and sepsis models is limited, as discussed above. Fourth, the teprenone data cited as the strongest experimental support for gastric-to-intestinal propagation within the GIA derive from a single research group and have not yet been independently replicated. External validation of these findings is needed to strengthen the evidence base for the GIA concept. These specific gaps represent priority areas for future investigation before the GIA framework can be translated into clinical practice.

### 8.3 Future directions and unanswered questions

Several critical questions remain to guide future research efforts, spanning mechanistic elucidation and clinical translation.

At the mechanistic level, the specific triggers driving HDAC5 upregulation in the gastric mucosa during sepsis remain unidentified. Additionally, the potential role of the gastric microbiome in modulating HDAC5 expression or ghrelin production warrants further investigation. Moreover, the concept of “ghrelin resistance”—analogous to insulin resistance in critical illness—requires systematic evaluation. Whether prolonged sepsis leads to the downregulation of ghrelin receptors or desensitization of downstream signaling pathways, and whether such changes could explain the failure of endogenous protective mechanisms, remains unclear. The optimal timing for ghrelin-axis restoration also remains unresolved; early intervention may prevent injury, whereas later intervention might promote repair, indicating distinct therapeutic windows.

Furthermore, interactions between the ghrelin axis and the gut microbiome, particularly its metabolites such as SCFAs and tryptophan derivatives, are incompletely understood. A comprehensive review by Benvenuto et al. recently highlighted the therapeutic potential of butyrate and other SCFAs in sepsis, emphasizing the need for further mechanistic studies to clarify their complex interplay with host immunity (225). In addition, variability in biomarker responses across different patient populations and insults, including trauma, sepsis, and coronavirus disease 2019, also requires further explanation, as do the long-term consequences of ICU dysbiosis on post-discharge outcomes, including susceptibility to recurrent infections and functional recovery.

Addressing these questions requires a multipronged research strategy. Preclinical studies should leverage conditional knockout models, including intestinal epithelial-specific *ATG2B* knockouts, ghrelin receptor knockouts, or PLK1 conditional mutants, to establish causal relationships. The role of the gastric microbiome should be investigated using germ-free and gnotobiotic models. Multi-kingdom analyses should extend beyond bacteria to include fungi, viruses, and bacteriophages, whose contributions remain largely unexplored. Finally, emerging concepts such as the gut–temperature axis warrant further investigation to understand how the microbiota modulates thermoregulation and whether this axis can be leveraged for personalized supportive care (29).

In the clinical setting, prospective observational cohorts should measure serial ghrelin, HDAC5 (in accessible cells such as peripheral blood mononuclear cells), and E2F1 levels in patients with trauma and sepsis to characterize their temporal dynamics and relationships with outcomes, such as AGI grade, nosocomial infection, and mortality. Precision medicine approaches should also identify genetic polymorphisms in GHRL, HDAC5, ATG2B, PLK1, or SIRT3 that may influence susceptibility to sepsis-induced AGI, thereby enabling the identification of high-risk patients who might benefit most from targeted interventions. Several of the preclinical interventions discussed in this review—including HDAC5 inhibitors, ghrelin supplementation, melatonin, and teprenone—represent promising candidates for GIA-directed therapy but remain at an early stage of translational development. Before clinical trials can be justified, several key steps must be completed: pharmacokinetic profiling in relevant large-animal models of trauma and sepsis, formal toxicology and safety pharmacology studies, establishment of optimal dosing regimens and therapeutic windows, and development of biomarker-based patient stratification strategies. A stepwise translational roadmap is proposed: (1) validation of target engagement and efficacy in trauma-specific preclinical models, including gastric mucosa-specific autophagy knockout models; (2) completion of the preclinical safety and dosing package required for regulatory approval; (3) Phase I/IIa proof-of-concept trials incorporating pharmacokinetic, safety, and biomarker endpoints in carefully selected patient cohorts with early AGI (grades I–II); and (4) definitive randomized controlled trials. None of the preclinical interventions discussed here has yet completed the full preclinical package required for an Investigational New Drug application and should therefore be regarded as future research directions rather than near-ready therapeutic options. This stepwise approach is consistent with the translational readiness tiers presented in **Supplementary Table 3**.

The development of ghrelin as a functional biomarker of GIA integrity, while conceptually attractive, faces substantial hurdles. No standardized clinical-grade assay currently exists; research studies rely on enzyme-linked immunosorbent assay (ELISA) or radioimmunoassay (RIA) kits with variable sensitivity and specificity for acylated (active) versus des-acyl ghrelin (161). Sample handling is demanding, requiring pre-chilled EDTA tubes with protease inhibitors and immediate centrifugation at 4 °C to prevent ghrelin degradation (94)—procedures that are difficult to implement in routine ICU practice. Furthermore, circulating ghrelin levels are profoundly influenced by nutritional status, feeding state, circadian rhythm, age, and body composition, complicating the interpretation of single time-point measurements (94). Reference ranges for critically ill populations have not been established, and the distinction between health-related variation and disease-related dysregulation remains unclear. Preclinical data support the concept that reduced ghrelin levels reflect impaired gastric autophagic function and increased susceptibility to intestinal barrier failure (18, 19), but the translation of these findings into a clinically useful biomarker will require the development of standardized assays, establishment of reference intervals in relevant patient populations, and prospective validation of ghrelin measurements against established markers of gastrointestinal dysfunction. These steps represent a necessary research agenda before ghrelin can be considered for biomarker-guided GIA assessment.

By advancing this integrated research agenda, the GIA concept can be translated from a mechanistic framework into actionable clinical strategies to improve outcomes in millions of patients affected by post-traumatic sepsis annually.

The GIA posits gastric autophagy as a putative regulatory node that, based on current evidence derived largely from intestinal epithelial and general sepsis models, is proposed to integrate diverse protective signals to preserve intestinal homeostasis. Direct validation of this gastric-centric mechanism in human post-traumatic sepsis remains a critical knowledge gap. Nevertheless, two lines of evidence—drawn primarily from the intestinal epithelium and systemic sepsis models—converge to support the proposed role of this node: the endogenous HDAC5–ghrelin–miR-143–ATG2B axis, which mediates physiological gastric–intestinal crosstalk, and pharmacologically inducible mechanisms, as exemplified by teprenone. Disruption of this axis may initiate a cascade from gastric injury to intestinal barrier failure, dysbiosis, and systemic inflammation, potentially contributing to post-traumatic sepsis.

Notably, the GIA concept extends beyond sepsis to encompass the entire post-traumatic continuum, suggesting that early gastric protection may mitigate late-onset septic complications. By framing gastric and intestinal dysfunction as interconnected processes, this paradigm provides a unified conceptual framework for biomarker discovery and the development of mechanism-based therapies. However, the GIA model remains a promising hypothesis that requires validation in prospective human cohorts and interventional studies before it can inform clinical practice. Future efforts should focus on addressing the key knowledge gaps identified in this review—including the need for direct evidence of gastric autophagy in human post-traumatic sepsis, trauma-specific biomarker validation, and rigorous assessment of the safety and efficacy of GIA-targeted interventions—to determine whether this framework can be translated into personalized strategies that improve outcomes in critically ill patients.

## Glossary

ICU: intensive care unit
GIA: Gastrointestinal Axis
HDAC5: histone deacetylase 5
ATG: autophagy-related gene
E2F1: E2F transcription factor 1
NF-κB: nuclear factor kappa-B
PLK1: polo-like kinase 1
mTOR: mammalian target of rapamycin
SIRT: sirtuin
I-FABP: intestinal fatty acid binding protein
AGI: acute gastrointestinal injury
VAP: ventilator-associated pneumonia
TJ: tight junction
JAM-A: junctional adhesion molecule A
CLP: cecal ligation and puncture
MLCK: myosin light chain kinase
IEC: intestinal epithelial cell
MUC2: mucin 2
SCFA: short-chain fatty acid
GPR: G-protein coupled receptor
IAA: indole-3-acetic acid
AHR: aryl hydrocarbon receptor
GALT: gut-associated lymphoid tissue
sIgA: secretory IgA
IAP: intestinal alkaline phosphatase
LPS: lipopolysaccharide
TLR4: Toll-like receptor 4
Lcn2: lipocalin-2
NGAL: neutrophil gelatinase-associated lipocalin
AKI: acute kidney injury
LC3: microtubule-associated protein 1A/1B-light chain 3
BECN1: beclin-1
ZO-1: zonula occludens-1
GHSR: growth hormone secretagogue receptor
SOD: superoxide dismutase
HSP: heat shock protein
WHO-ORS: World Health Organization oral rehydration solution
MDA: malondialdehyde
IL: interleukin
HPA: heparanase
STING: stimulator of interferon gene
RIPK3: receptor-interacting protein kinase 3
MLKL: mixed lineage kinase domain-like protein
TBK1: TANK-binding kinase 1
IRF: interferon regulatory factor
GPX: glutathione peroxidase
DAMP: damage-associated molecular pattern
OMV: outer membrane vesicle
SOFA: sequential organ failure assessment
ALI: acute lung injury
Wnt: wingless-related integration site
CCL1: C-C motif chemokine ligand 1
HMGB1: high mobility group box 1
ENS: enteric nervous system
Mn-CD: manganese-carbon dot
IPA: indole-3-propionic acid
NaB: sodium butyrate
TMAO: trimethylamine N-oxide
ESICM: European Society of Intensive Care Medicine
GI: gastrointestinal
TFF: trefoil factor family
MODS: multiple organ dysfunction syndrome
APACHE II: acute physiology and chronic health evaluation II
EEN: early enteral nutrition
RCT: Randomized controlled trial
ESPEN: European Society for Clinical Nutrition and Metabolism
PN: parenteral nutrition
TPN: total parenteral nutrition
GLN: glutamine
REDOXS: REducing Deaths due to OXidative Stress
RE-ENERGIZE: RE-Evaluating the Efficacy of ENteral ERGlutamine in Thermal Injury
TBSA: total body surface area
PROSPECT: Probiotics: Prevention of Severe Pneumonia and Endotracheal Colonization Trial
CDI: Clostridium difficile infection
PROPATRIA: Probiotics in Pancreatitis Trial
AI: artificial intelligence
FMT: fecal microbiota transplantation
Pi-PEG: phosphorylated polyethylene glycol.
