# Supplementary material for: Gastrointestinal axis in post-traumatic sepsis: from molecular mechanisms to translational perspectives
Source: Front Immunol. 2026 Jul 17;17:1855955. doi: 10.3389/fimmu.2026.1855955 (PMC13423654; doi:10.3389/fimmu.2026.1855955)
Supplement: Supplementary file 1 [file Supplementaryfile1.docx]

Supplementary Material

# Supplementary Tables

**Supplementary Table S1. Full search strategy and yield for each database**

| **Database** | **Search Strategy** | **Records Retrieved** |
| --- | --- | --- |
| **PubMed** | (“trauma”[tiab] OR “burn”[tiab] OR “shock”[tiab] OR “sepsis”[tiab] OR “post-traumatic”[tiab]) AND (“gastrointestinal”[tiab] OR “stomach”[tiab] OR “gastric”[tiab] OR “intestine”[tiab] OR “gut barrier”[tiab] OR “autophagy”[tiab]) AND (“microbiome”[tiab] OR “microbiota”[tiab] OR “biomarker”[tiab] OR “therapy”[tiab] OR “treatment”[tiab]) | 9,210 |
| **Web of Science** | TS = (“trauma” OR “burn” OR “shock” OR “sepsis” OR “post-traumatic”) AND TS = (“gastrointestinal” OR “stomach” OR “gastric” OR “intestine” OR “gut barrier” OR “autophagy”) AND TS = (“microbiome” OR “microbiota” OR “biomarker” OR “therapy”) | 3,980 |
| **Scopus** | TITLE-ABS-KEY(“trauma” OR “burn” OR “shock” OR “sepsis”) AND TITLE-ABS-KEY(“gastrointestinal” OR “stomach” OR “gastric” OR “intestine” OR “gut barrier” OR “autophagy”) AND TITLE-ABS-KEY(“microbiome” OR “microbiota” OR “biomarker” OR “therapy”) | 27,445 |

**Notes:** All searches were limited to records published from database inception to December 31, 2025. PubMed searches used [tiab] field tags to restrict retrieval to titles and abstracts for maximum precision. Web of Science searches used TS= (Topic) field tags, which search titles, abstracts, and keywords. Scopus searches used TITLE-ABS-KEY field tags, which search titles, abstracts, and keywords. No language restrictions were applied during the search. The marked difference in yield between Scopus and PubMed is attributable to Scopus's broader disciplinary coverage, including substantial content in physical sciences, engineering, and social sciences, where the search terms appeared in non-clinical contexts. Despite these differences, using all three databases ensured comprehensive coverage of the relevant biomedical literature. Records retrieved represent the initial search yield; after deduplication and relevance-based screening, studies were selected for inclusion as described in Section 2.2.

**Supplementary Table S2. Evidence classification by study type, disease model, evidence strength, and relevance to post-traumatic sepsis**

| **Pathway / Finding** | **Key Reference(s)** | **Study Type** | **Disease Model** | **Evidence Strength** | **Relevance to Post-Traumatic Sepsis** | **Validated in Human Post-Traumatic Sepsis?** |
| --- | --- | --- | --- | --- | --- | --- |
| **HDAC5–ghrelin–miR-143–ATG2B axis** |  |  |  |  |  |  |
| Ghrelin protects small intestinal epithelium via enhancing autophagy | Wan et al. (2016) (17) | Original research | Rat CLP model | Indirect—general sepsis model | High: ghrelin-mediated autophagy is central to GIA; CLP model shares features with sepsis but lacks trauma-specific hypoperfusion | No |
| Ghrelin improves small intestinal barrier via miR-143/ATG2B-mediated autophagy | Liu et al. (2025) (18) | Original research | Rat CLP + LPS-treated IEC-6 cells | Indirect—general sepsis model | High: defines molecular mechanism of ghrelin-mediated barrier protection | No |
| HDAC5 promotes intestinal sepsis via Ghrelin/E2F1/NF-κB axis | Li et al. (2021) (19) | Original research | Human sepsis intestinal tissue + mouse CLP | Mixed: human tissue data + murine model | High: human intestinal HDAC5 upregulation directly observed in sepsis patients | Partially—human tissue data available, but not trauma-specific |
| Ghrelin/GHSR axis induces M2 macrophage polarization and alleviates intestinal barrier dysfunction | Zhu et al. (2023) (95) | Original research | Rat sepsis model | Indirect—general sepsis model | Moderate: extends understanding of ghrelin immunomodulation | No |
| **PLK1–mTOR pathway** |  |  |  |  |  |  |
| PLK1–mTOR axis regulates autophagy to prevent intestinal barrier dysfunction | Cao et al. (2023) (97) | Original research | Conditional PLK1 knock-in mice + LPS-treated IEC cells | Indirect—general sepsis model | Moderate: identifies parallel autophagy regulatory node | No |
| **SIRT3 pathway** |  |  |  |  |  |  |
| Melatonin upregulates SIRT3, attenuates sepsis-induced small-intestine injury | Xu et al. (2021) (98) | Original research | Murine CLP + endotoxemia + intestinal-specific SIRT3 KO mice | Indirect—general sepsis model | Moderate: mitochondrial protection pathway; genetic evidence strengthens mechanistic confidence | No |
| **Teprenone (geranylgeranylacetone)** |  |  |  |  |  |  |
| Teprenone oral rehydration solution improves survival in burn shock | Liu et al. (2025) (21) | Original research | Rat severe burn model | Direct—trauma/burn-specific model | Very high: burn injury is a clinically relevant form of trauma; directly tests gastric protection in GIA-relevant context | No—but burn model is clinically proximal |
| Bibliometric analysis and teprenone efficacy in early post-burn shock | Liu et al. (2025) (22) | Original research | Rat burn shock model | Direct—trauma/burn-specific model | Very high: systematic comparison of gastroprotective agents in trauma context | No |
| **Heparanase (HPA)** |  |  |  |  |  |  |
| Heparanase inhibition improves acute gastrointestinal injuries in sepsis | Chen et al. (2023) (107) | Original research | Rat sepsis model | Indirect—general sepsis model | Moderate: glycocalyx protection mechanism | No |
| **Spermidine** |  |  |  |  |  |  |
| Spermidine improves gut barrier integrity and microbiota in diet-induced obese mice | Ma et al. (2020) (108) | Original research | Murine diet-induced obesity model | Extrapolated—non-sepsis, non-trauma context | Low: model context (obesity) differs substantially from post-traumatic sepsis | No |
| **Necroptosis–STING crosstalk** |  |  |  |  |  |  |
| RIPK3-MLKL necroptotic signalling amplifies STING pathway and exacerbates sepsis | Zhang et al. (2023) (109) | Original research | Murine sepsis models | Indirect—general sepsis model | Moderate: cell death–inflammation interface relevant to GIA | No |
| **Ferroptosis** |  |  |  |  |  |  |
| Ghrelin and Ferrostatin-1 protect against sepsis-induced intestinal ferroptosis | Hou et al. (2024) (110) | Original research | Murine CLP model | Indirect—general sepsis model | Moderate: extends ghrelin's protective mechanisms to ferroptosis inhibition | No |
| **Gut–lymph hypothesis** |  |  |  |  |  |  |
| Gut-derived mesenteric lymph increases endothelial permeability and promotes lung injury after hemorrhagic shock | Magnotti et al. (1998) (12) | Original research | Rat hemorrhagic shock model | Direct—trauma/shock-specific model | Very high: hemorrhagic shock directly relevant to post-traumatic hypoperfusion | No |
| Post-hemorrhagic shock mesenteric lymph is cytotoxic to endothelial cells and activates neutrophils | Upperman et al. (1998) (13) | Original research | Rat hemorrhagic shock model | Direct—trauma/shock-specific model | Very high | No |
| **Early enteral nutrition (EEN)** |  |  |  |  |  |  |
| EEN reduces mortality and improves outcomes in major burn injury: meta-analysis | Pu et al. (2018) (172) | Meta-analysis of RCTs | Human burn patients | Direct—human trauma/burn evidence | Very high: meta-analysis of RCTs in burn patients provides strongest clinical evidence for GIA-targeted intervention | Yes—multiple RCTs |
| EEN for patients with sepsis or septic shock: systematic review | Grillo-Ardila et al. (2024) (173) | Systematic review | Human sepsis patients | Indirect—general sepsis | High: supportive evidence from broader sepsis population | Yes |
| **Glutamine (GLN)** |  |  |  |  |  |  |
| GLN sustains energy metabolism and alleviates liver injury in burn sepsis via SIRT4 | Yang et al. (2024) (190) | Original research | Murine burn sepsis model | Direct—burn sepsis model | High: burn sepsis model directly relevant | No |
| GLN maintains intestinal mucus barrier in burned septic mice | Wu et al. (2023) (191) | Original research | Murine burn sepsis model | Direct—burn sepsis model | High: directly relevant | No |
| REDOXS trial: glutamine and antioxidants in critically ill patients | Heyland et al. (2013) (194) | Multicenter RCT | Human critically ill patients | Direct—human clinical evidence | Very high: large RCT in critically ill population | Yes |
| RE-ENERGIZE trial: enteral glutamine for burn injuries | Heyland et al. (2022) (195) | Multicenter RCT | Human burn patients | Direct—human trauma/burn evidence | Very high: RCT specifically in burn patients | Yes |

**Notes:** **Evidence strength categories:** **Direct**—trauma/burn-specific model or human evidence: Findings from burn, trauma, hemorrhagic shock models, or human trauma/burn cohorts. **Indirect**—general sepsis model: Findings from CLP, LPS, or general sepsis models without a trauma-specific component. **Extrapolated**—non-sepsis, non-trauma context: Findings from disease models substantially different from post-traumatic sepsis (e.g., obesity). All reference numbers correspond to the main manuscript reference list.

**Supplementary Table S3. Evidence-ranking table for key GIA pathways, biomarkers, and therapeutic interventions**

**Part A: Key molecular pathways**

| **Pathway** | **Source of Evidence** | **Human Data Available?** | **Validated in Post-Traumatic Sepsis?** | **Translational Readiness** |
| --- | --- | --- | --- | --- |
| HDAC5–ghrelin–E2F1–NF-κB axis | Animal experiments (CLP, LPS) + human tissue (HDAC5 expression in sepsis patients) | Partially (human intestinal tissue) | No | Tier 3—promising preclinical |
| PLK1–mTOR–autophagy | Animal experiments (conditional KI mice) + cell culture (IEC) | No | No | Tier 3—promising preclinical |
| SIRT3–mitochondrial protection | Animal experiments (CLP, endotoxemia, conditional KO mice) | No | No | Tier 3—promising preclinical |
| Necroptosis–STING crosstalk | Animal experiments (murine sepsis) | No | No | Tier 4—emerging |
| Ghrelin–ferroptosis inhibition | Animal experiments (murine CLP) | No | No | Tier 4—emerging |

**Part B: Biomarkers**

| **Biomarker** | **Source of Evidence** | **Human Data Available?** | **Validated in Post-Traumatic Sepsis?** | **Translational Readiness** |
| --- | --- | --- | --- | --- |
| I-FABP | Clinical studies + meta-analyses | Yes | Yes (trauma and sepsis cohorts) | Tier 2—clinical evidence available; standardization needed |
| D-lactate | Clinical studies | Yes | Partially (sepsis cohorts; limited trauma-specific data) | Tier 2—clinical evidence available |
| Citrulline | Clinical studies | Yes | Partially (ICU cohorts; limited trauma-specific data) | Tier 2—clinical evidence available |
| AGI grading | Multicenter prospective study | Yes | Yes (general ICU; not trauma-specific) | Tier 2—clinical evidence available |
| Ghrelin (as functional GIA biomarker) | Mechanistic hypothesis + animal data | No | No | Tier 4—emerging concept |
| Microbiome signatures | Clinical studies | Yes | Partially (ICU cohorts; trauma-specific data emerging) | Tier 3—promising; clinical feasibility challenges |
| SCFAs | Clinical studies | Yes | Partially (limited trauma-specific data) | Tier 3—promising |

**Part C: Therapeutic interventions**

| **Intervention** | **Source of Evidence** | **Human Data Available?** | **Validated in Post-Traumatic Sepsis?** | **Translational Readiness** |
| --- | --- | --- | --- | --- |
| Early enteral nutrition (EEN) | Meta-analyses of RCTs | Yes | Yes (burn and trauma RCTs) | Tier 1—guideline-supported |
| Glutamine (severe burns) | RCTs + meta-analyses | Yes | Yes (burn RCTs; controversial in general ICU) | Tier 1 (burns) / Tier 2 (general sepsis) |
| Probiotics | RCTs + meta-analyses | Yes | Partially (mixed results; safety concerns in ICU) | Tier 2—clinical evidence with safety caveats |
| Synbiotics | RCTs | Yes | Partially | Tier 2—clinical evidence available |
| Postbiotics (SCFAs) | Animal experiments + limited clinical studies | Limited | No | Tier 3—promising preclinical |
| Fecal microbiota transplantation (FMT) | Case reports + animal experiments | Limited (case reports only) | No | Tier 4—experimental in ICU |
| HDAC5 inhibitors (LMK-235) | Animal experiments (murine sepsis) | No | No | Tier 4—emerging |
| Ghrelin supplementation | Animal experiments (CLP, hemorrhagic shock) | No | No | Tier 3—promising preclinical |
| Teprenone | Animal experiments (burn shock) | No | Direct evidence in burn model | Tier 3—promising preclinical |
| Melatonin (SIRT3 activation) | Animal experiments (CLP, endotoxemia) | No | No | Tier 3—promising preclinical |
| Spermidine | Animal experiments (diet-induced obesity) | No | No (non-sepsis model) | Tier 4—emerging |
| HPA inhibition (LMWH) | Animal experiments (rat sepsis) | No | No | Tier 4—emerging |
| Pi-PEG | Animal experiments | No | No | Tier 4—emerging |

**Translational Readiness Tiers:**

- **Tier 1:** Guideline-supported or evidence-based; supported by multiple RCTs or meta-analyses in relevant populations.
- **Tier 2:** Promising with some clinical evidence; human data available but further validation or standardization required.
- **Tier 3:** Promising preclinical strategy; compelling mechanistic rationale and animal model evidence; no human data yet.
- **Tier 4:** Emerging/experimental; limited in vivo validation or extrapolated from non-sepsis contexts; requires extensive further study before clinical translation.

**Supplementary Table S4. Emerging organ axes involving the gut in sepsis**

| **Axis** | **Key mechanisms** | **Clinical implications** |
| --- | --- | --- |
| Gut–temperature | Gut microbiota (e.g., *Lachnospiraceae*) modulates body temperature trajectories (29) | May enable personalized thermoregulatory support |
| Gut–muscle | Reduced SCFA production impairs mitochondrial function; uremic toxins promote muscle atrophy (27, 28) | Microbiota-targeted therapies may improve functional recovery |
| Gut–kidney | Gut-derived uremic toxins (indoxyl sulphate, p-cresyl sulphate) contribute to AKI; AKI exacerbates gut barrier dysfunction (26) | Targeting gut–kidney axis may provide novel AKI therapies |
| Gut–heart | TMAO promotes atherosclerosis; SCFAs regulate blood pressure; dysbiosis-associated inflammation contributes to myocardial dysfunction (50) | Microbiota modulation may reduce cardiovascular events in sepsis survivors |

*SCFA* short-chain fatty acid; *AKI* acute kidney injury; *TMAO* Trimethylamine N-oxide
